# Advances in traditional Chinese medicine for modulating macrophage polarization in the treatment of rheumatoid arthritis

**DOI:** 10.3389/fphar.2026.1702947

**Published:** 2026-09-15

**Authors:** Wenjiao Wang, Jianhui Ma, Xudong Zhang, Huimin Mei, Lu Wang, Jinping Yu, Songmiao Xu, Cong Huang

**Affiliations:** 1 Qiannan Traditional Chinese Medicine Hospital, Duyun, Guizhou, China; 2 Guizhou University of Traditional Chinese Medicine, Guiyang, China; 3 National Key Laboratory of Lead Drugability Research, China State Institute of Pharmaceutical Industry, Shanghai, China; 4 Shenzhen Bao’an Authentic TCM Therapy Hospital, Shenzhen, Guangdong, China

**Keywords:** inflammatory responses, macrophage polarization, metabolic reprogramming, rheumatoid arthritis, traditional Chinese medicine

## Abstract

Rheumatoid arthritis (RA) is a chronic autoimmune disorder characterized by persistent synovitis and progressive joint destruction, wherein macrophage polarization constitutes a critical pathogenic mechanism governing both the initiation and perpetuation of synovial inflammation. The conventional M1 and M2 dichotomy has evolved into a continuum model encompassing disease-stage-specific synovial subsets, including remission-associated MerTK-positive populations and pro-inflammatory clusters, reflecting the functional heterogeneity of macrophages in the rheumatoid microenvironment. Modulating macrophage polarization accordingly represents a promising therapeutic strategy; however, pharmacological agents specifically designed to target macrophage phenotypes remain in nascent stages of development. Traditional Chinese medicine (TCM) has emerged as a potential avenue for addressing this therapeutic gap. Extensive experimental evidence has demonstrated that diverse multi-botanical drug formulations and purified bioactive metabolites, including sinomenine, icariin, and curcumin, can effectively modulate macrophage polarization through interconnected pathways involving NF-κB, JAK-STAT, and PI3K-Akt signaling. These interventions further act through metabolic reprogramming between glycolysis and oxidative phosphorylation, operating *via* metabolite-driven epigenetic modifications such as histone lactylation and the IRG1-itaconate-TET2 axis, alongside NLRP3 inflammasome restriction and autophagic regulation, all of which collectively promote the rebalancing of inflammatory and reparative macrophage phenotypes. This review systematically consolidates current mechanistic understanding and critically assesses translational gaps, including the predominance of rodent collagen-induced arthritis (CIA) models, the scarcity of human synovial macrophage validation, undefined pharmacokinetics of active metabolites, and the need for patient stratification by synovial subset markers. It aims to establish a theoretical framework facilitating future investigations into precise molecular targets and supporting the rational development of macrophage-targeted, clinically viable interventions for RA.

## 1 Introduction

Rheumatoid Arthritis(RA) is a chronic, systemic autoimmune disorder characterized by persistent synovitis, leading to joint pain, swelling, stiffness, and functional impairment. It represents one of the most prevalent autoimmune rheumatic diseases among the elderly population worldwide. The global incidence of RA is approximately 40 per 100,000 person-years, with a prevalence of around 0.5% (Radu and Bungau, 2021; Brown et al., 2024). The mean age of onset typically ranges from 40 to 60 years, and the female-to-male ratio varies between 2:1 and 3:1 (Jang et al., 2022; Ng et al., 2014). According to 2016 data, RA was the most common autoimmune rheumatic disease in South Korea, with 96,330 recorded cases (188.5 per 100,000 population) (Kim et al., 2020). In the United States, its prevalence ranges from 0.5% to 1% (Di Matteo et al., 2023). Meanwhile, in China, between 1990 and 2019, there was an increasing trend in RA incidence, prevalence, mortality, and disability-adjusted life years (DALYs) (Zhao et al., 2024). Poorly controlled RA is associated with reduced life expectancy and an approximately two-fold increased risk of malignancies and cardio-vascular diseases compared to well-managed cases (Turesson, 2013). Therefore, early diagnosis and intervention are critical for preventing disease progression and improving clinical outcomes in RA patients (Ain et al., 2019). For the convenience of literature sorting, we adopt the classical M1/M2 nomenclature in this study. Nevertheless, macrophage polarization is actually a dynamic and continuous spectrum rather than discrete subtypes.

In recent years, Macrophage polarization has been found to be an important mechanism in the development of RA, emerging evidence has demonstrated that macrophages can undergo phenotypic plasticity, adapting their functionality to varying tissue milieus. Although the spectrum of macrophage activation states is broad and context-dependent, two primary polarization extremes are widely recognized: classically activated (M1) macrophages and alternatively activated (M2) macrophages. These subsets represent divergent functional programs, with M1 macrophages typically promoting inflammation and M2 macrophages associated with immunoregulation and tissue repair. As antigen-presenting cells, macrophages contribute to immune activation by presenting non-self antigens to naïve T cells and secreting cytokines and growth factors, thereby inducing either Th1- or Th2-mediated immune responses (Ross et al., 2021). A growing body of evidence suggests that while M1 and M2 macrophages represent extremes of a polarization spectrum, in reality, macrophages often exhibit intermediate phenotypes involved in immunoregulation or tissue repair. These intermediate states are defined by distinct metabolic pathways, surface markers, and cytokine profiles. Consequently, research on macrophage polarization has evolved from initial phenomenological observations to indepth investigations into its molecular mechanisms and pathological significance across various diseases. Notably, macrophage polarization has been implicated in the pathogenesis of rheumatoid arthritis (RA), where skewed polarization states contribute to disease progression and chronic inflammation.

Although modern biomedical research is steadily unraveling the molecular basis of inflammatory diseases, traditional medical systems such as Traditional Chinese Medicine (TCM) offer a holistic approach rooted in centuries of clinical experience and observation. TCM employs complex botanical drugs and metabolites, which contain numerous bioactive metabolites capable of modulating inflammatory responses through multiple pathways (Yang et al., 2023). In recent years, a growing body of evidence has demonstrated the potential of TCM to regulate macrophage polarization, thereby offering novel therapeutic strategies for RA. Nevertheless, a comprehensive review synthesizing the role of TCM in modulating macrophage polarization in the context of inflammatory diseases remains lacking. This article aims to address this gap by consolidating and analyzing current research on the topic. We have conducted an extensive review of both international and domestic studies concerning the interplay among TCM, macrophage polarization, and RA. Given our focus on TCM and its regulatory effects on macrophage polarization in RA, a considerable portion of relevant literature is published in Chinese. Consequently, we have incorporated a significant number of Chinese references. This review provides readers with an in-depth understanding of the mechanisms through which TCM modulates macrophage polarization and offers a comprehensive analysis of the TCM-macrophage polarization-RA triad.

## 2 The role of macrophage polarization in rheumatoid arthritis

### 2.1 Concept of macrophage polarization

Early investigations primarily focused on the role of macrophages in immune defense. However, with technological advances, the understanding of macrophage polarization has significantly expanded. It is now well-established that, depending on microenvironmental stimuli, macrophages can polarize into classically activated, pro-inflammatory M1 phenotypes or alternatively activated, anti-inflammatory M2 phenotypes. An imbalance between these polarization states is closely associated with the pathogenesis of various diseases (Callegari et al., 2023).

Macrophages reside in diverse tissues, giving rise to specialized populations that perform tissue-specific and niche-specific functions. For instance, macrophages in the central nervous system, known as microglia, are involved in debris clearance, synaptic remodeling, and immune surveillance. In adipose tissue, adipose-associated macro-phages modulate signaling related to insulin sensitivity. Alveolar macrophages phagocytose inhaled foreign particles and clear excess pulmonary surfactant. Kupffer cells, or hepatic macrophages, contribute to the clearance of microbes, red blood cells, and cellular debris from the blood. Bone marrow macrophages support erythropoiesis by engulfing extruded nuclei from developing erythrocytes. In the spleen, various macrophage subsets-including marginal zone, red pulp, and white pulp macrophages-participate in immune surveillance, iron metabolism, and clearance of apoptotic cells (Funes et al., 2018).

M1 macrophages can be induced by lipopolysaccharide (LPS) alone or in combi-nation with cytokines such as interferon-gamma (IFN-γ), tumor necrosis factor-alpha (TNF-α), or granulocyte-macrophage colony-stimulating factor (GM-CSF). These cells are characterized by the secretion of pro-inflammatory cytokines-including interleukin such as IL-12, IL-1β, IL-6, IL-23, and TNF-α-and the production of inducible nitric oxide synthase (iNOS)-dependent reactive oxygen intermediates (ROI) and reactive nitrogen intermediates (RNI). Although M1 macrophages are essential for host defense against pathogens, sustained M1 activation and the consequent release of inflammatory mediators can lead to tissue damage.

In contrast, M2 polarization is promoted by stimuli such as IL-4, IL-13, IL-10, transforming growth factor-beta (TGF-β), glucocorticoids, and immune complexes. M2 macrophages are defined by the expression of markers including arginase-1 (Arg1), chitinase-like protein 3 (Chil3/Ym1), resistin-like molecule alpha (Relmα/Fizz1), man-nose receptor (MRC1/CD206), and the anti-inflammatory cytokine IL-10. This phenotype is associated with anti-inflammatory responses, promotion of angiogenesis, and tissue repair (Wang et al., 2014).

Macrophage polarization is governed by a complex and multifaceted mechanism involving the interplay of numerous signaling pathways, transcription factors, and metabolic processes. The underlying mechanisms of macrophage polarization in inflammatory diseases, however, vary considerably. For instance, in chronic obstructive pulmonary disease (COPD), cigarette smoke exposure induces human bronchial epithelial cells to secrete exosomes containing miR-221-3p, which targets SOCS3 and modulates the STAT3 signaling pathway, thereby promoting M1 macrophage polarization and exacerbating inflammatory responses. Conversely, in other contexts such as infection with Thelazia callipaeda, T.cp-MIF induces macrophage polarization *via* the C/EBPβ–SESN2 axis, leading to M2b polarization and subsequent suppression of inflammation (Jia et al., 2024; Luo et al., 2025).

Macrophage polarization represents a core manifestation functional plasticity, and its conceptual framework has evolved from the traditional M1/M2 dichotomy toward a complex dynamic networks. These findings illustrate that macrophage polarization is regulated by di-verse factors across different pathological environments, highlighting both the complexity and context-dependency of its mechanisms. A deeper understanding of these regulatory networks is therefore essential for the development of targeted therapeutic strategies.

### 2.2 Molecular mechanisms of macrophage polarization

Macrophage functional plasticity is central to immune defense tissue homeostasis and repair processes primarily manifested through the dynamic balance between pro-inflammatory M1 and anti-inflammatory reparative M2 phenotypes. This process is precisely regulated by multi-layered molecular networks. Key signaling pathways such as TLR/NF-kB JAK/STAT and PPARs receive and integrate microenvironmental signals directly driving macrophage polarization. Epigenetic mechanisms including DNA methylation histone modifications and non-coding RNAs fine-tune gene expression at transcriptional and post-transcriptional levels granting cellular plasticity and long-term memory. Meanwhile metabolic reprogramming provides energy and biosynthetic precursors for polarization—M1 macrophages favor glycolysis and the pentose phosphate pathway while M2 types rely on OXPHOS and FAO. Metabolic intermediates also act as signaling molecules and epigenetic regulators further shaping immune phenotypes. These mechanisms intersect synergize or antagonize collectively forming a sophisticated regulatory network for polarization.

#### 2.2.1 Key signaling pathways of macrophage polarization

Macrophage polarization is governed by a complex signaling network that balances pro-inflammatory M1 and anti-inflammatory reparative M2 phenotypes. M1 polarization is primarily driven by the TLR/NF-κB axis: pathogen- or damage-associated molecular patterns activate MyD88-dependent signaling, leading to NF-κB-mediated pro-inflammatory cytokine production. IFN-γ triggers JAK/STAT1, upregulating iNOS, CD86, and ROS; KLF9 further promotes M1 *via* this pathway (Xu et al., 2025). CCL5 enhances M1 through CCR1/CCR5-mediated MAPK and NF-κB activation (Liu et al., 2019), while HIF-1α stabilization under hypoxia boosts glycolysis and IL-1β release (Zhao et al., 2025). M2 polarization is mainly induced by IL-4/IL-13 *via* STAT6, which induces Arg1 and other anti-inflammatory genes (Byles et al., 2013). USP25 stabilizes STAT6, whereas TRAF3 regulates its ubiquitination. PPARγ and PPARδ act as lipid sensors that enhance fatty acid oxidation and anti-inflammatory mediator production (Shi et al., 2023).

KLF4 serves as a core M2 regulator cooperating with PPARγ, and TREM2 facilitates M1-to-M2 transition by suppressing PI3K/AKT/NF-κB signals and downregulating CXCL3 (Fang et al., 2024). Multilayered crosstalk includes PPARγ dephosphorylation at T166 promoting reparative factors *via* STAT3 and a HIF-1α–glycolysis positive feedback loop that sustains M1 inflammation. Immune checkpoints also participate: IL-4/STAT6-induced FcγRIIB mediates PD-1 therapy resistance, and Siglec-10 acts on HIF-1α to foster immunosuppression (Zhang J. et al., 2024). Interventions targeting these nodal points, such as nanodrugs modulating TLR/STAT signaling or regulating HIF-1α, offer novel therapeutic avenues.

Thus, the coordinated regulation by STAT, NF-κB, PPAR, and other signaling pathways dictates immune homeostasis and underpins the development of macrophage-targeted nanomedicine and metabolic therapies. M1 polarization is primarily driven by the TLR/NF-κB and IFN-γ/STAT1 pathways, and further modulated by factors such as KLF9, the CCL5/CCR1/CCR5–MAPK/NF-κB axis, and HIF-1α, which collectively promote pro-inflammatory responses. Conversely, M2 polarization relies on the IL-4/IL-13/STAT6 pathway—tightly regulated by USP25 and TRAF3—along with PPARs activated by KLF4. Additionally, TREM2 facilitates M2 transition by suppressing the PI3K/AKT/NF-κB pathway. Through synergistic or antagonistic interactions among these networks, the M1/M2 balance is dynamically regulated, thereby influencing inflammatory responses, tissue repair, and disease progression. Understanding these molecular switches offers valuable insights for designing macrophage-centered immunotherapies against chronic inflammatory diseases and metabolic disorders. The core signaling cascade governing M1/M2 macrophage polarization is summarized in Figure 1.

**FIGURE 1:**
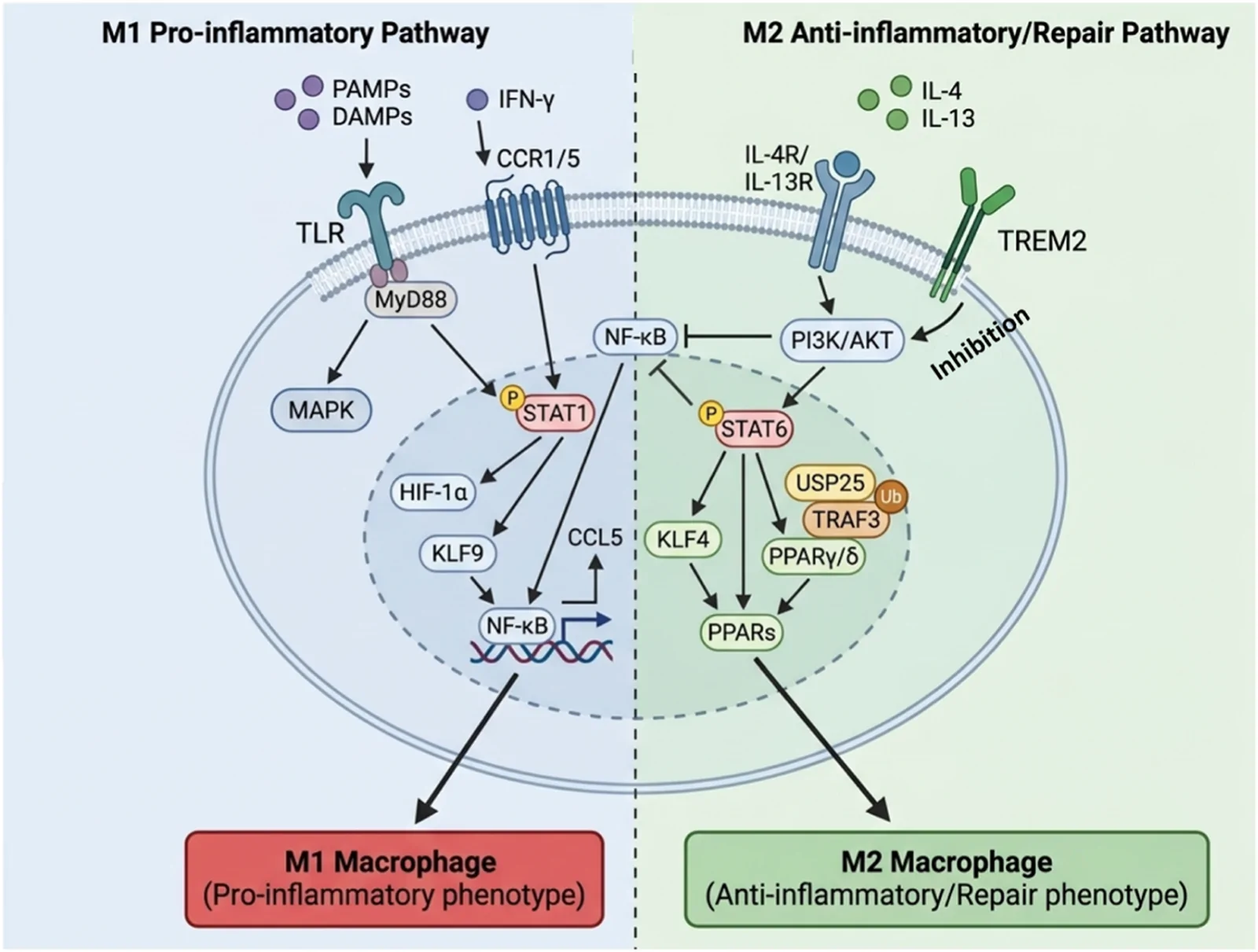
Macrophage polarization signaling pathway network. Schematic diagram of core signaling pathways controlling M1 pro-inflammatory and M2 anti-reparative macrophage polarization. Pathogen/damage-associated molecular patterns (PAMPs/DAMPs) and IFN-γ initiate TLR-MyD88-NF-κB/MAPK-STAT1 cascades to drive M1 polarization, while IL-4/IL-13 activate IL-4R/IL-13R-dependent STAT6 signaling to induce M2 polarization. Transcription factors including KLF9, CCL5, KLF4, PPARγ/δ, and regulators USP25/TRAF3 coordinate the phenotypic switch, and TREM2 exerts inhibitory effects on NF-κB to facilitate M1-to-M2 transition.

#### 2.2.2 Epigenetic regulation of macrophage polarization

Macrophage polarization is epigenetically orchestrated by DNA methylation, histone modifications, and non-coding RNAs, which dynamically modulate plasticity in diseases such as atherosclerosis, cancer, and inflammatory disorders. DNA methylation: Loss of TET2 elevates Dusp10 promoter methylation, suppressing Dusp10 and enhancing JNK phosphorylation, which promotes BRCC3-mediated NLRP3 deubiquitination and inflammasome activation, accelerating atherosclerosis. Impaired serine metabolism reduces H3K27 trimethylation, upregulating IGF1 and activating the p38–JAK/STAT1 pathway to drive M1 and inhibit M2 polarization (Shan et al., 2022). DNMT3A mutations cooperate with TET2 to alter methylation patterns and amplify inflammatory macrophage phenotypes (Rauch et al., 2023). Conversely, low-dose decitabine demethylates KLF4 and PPARγ promoters, promoting their binding and M2 anti-inflammatory gene expression. Uhrf1-dependent hypermethylation of the TNF-α promoter, controlled by PPARγ, inhibits necroptosis. Thus, DNA methylation precisely tunes key inflammatory genes (Mei et al., 2023).

Histone modifications: Lactylation serves as a metabolism-epigenetics bridge. MCT4 deficiency causes lactate accumulation, inducing H3K18 lactylation that activates repair genes, promoting M2 polarization and suppressing atherosclerosis (Zhang Y. et al., 2024). In the tumor microenvironment, histone lactylation upregulates YTHDF2, which degrades PER1 and TP53 mRNA to drive tumorigenesis. Acetylation: HDAC6 deacetylates FoxO1, enhancing its phosphorylation and M2 polarization in periodontitis (Yu et al., 2021); the HDAC2/SP1 axis remodels H3 acetylation to foster M2-like tumor-associated macrophages (Zheng et al., 2023). SETDB2 deficiency reduces H3K9 methylation, impairing gene silencing and exacerbating inflammation. Hence, histone modifications integrate metabolic and inflammatory signals (Zhang et al., 2021).

Non-coding RNAs: The lncRNA LARRPM recruits TET1 to demethylate the LINC00240 promoter, suppressing tumor progression, while reducing TET1 at the CSF1 promoter to promote methylation and inhibit M2 polarization (Huang et al., 2023). Exosomal ncRNAs mediate intercellular communication: colorectal cancer-derived miR-106a-5p targets SOCS6 to activate JAK2/STAT3 and drive M2; breast cancer exosomal lncRNA HAGLROS promotes M2 *via* the miR-135b-3p/COL10A1 axis (Meng et al., 2024). miR-34a inhibits M2 by suppressing KLF4 (Xia et al., 2020). Thus, lncRNAs exosomal lncRNAs and modified miRNAs form a multi-level regulatory network that precisely controls macrophage polarization making them crucial regulatory factors and therapeutic targets in inflammation and cancer.

In summary, these three epigenetic mechanisms collectively fine-tune macrophage plasticity and offer therapeutic targets at the metabolism-epigenetics interface for atherosclerosis, cancer, and inflammatory disease. MMetabolic reprogramming is central to macrophage polarization. Multi-layered epigenetic mechanisms orchestrating macrophage functional plasticity are illustrated in Figure 2.

**FIGURE 2:**
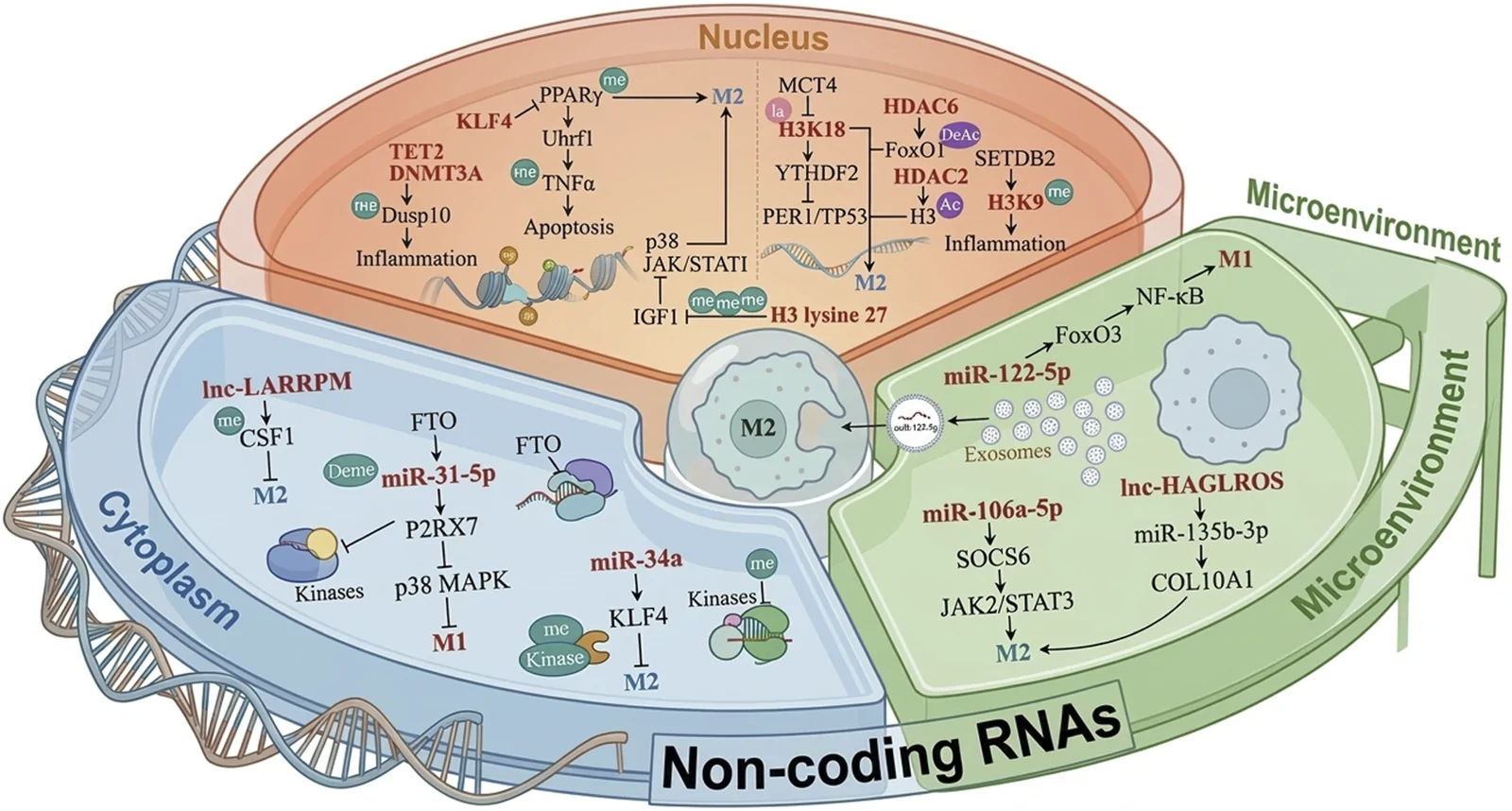
Epigenetic regulatory network of macrophage polarization. Three major epigenetic layers are integrated: (1) DNA methylation mediated by DNMT3A and TET2 modulating transcription of KLF4, TNF-α and Dusp10 *via* Uhrf1; (2) histone modifications including H3K18 lactylation, histone acetylation/deacetylation (HDAC2/6) and H3K9 trimethylation (SETDB2); (3) non-coding RNAs (lncRNA LARRPM/HAGLROS, miR-34a, miR-106a-5p, miR-135b-3p) and exosomal ncRNAs mediating intercellular communication to balance M1/M2 polarization.

#### 2.2.3 Metabolic reprogramming as a core driver of Macrophage polarization

Metabolic reprogramming is central to macrophage functional plasticity. M1 pro-inflammatory macrophages rely on glycolysis and the pentose phosphate pathway (PPP), whereas M2 anti-inflammatory/reparative cells favor oxidative phosphorylation (OXPHOS) and fatty acid oxidation (FAO). Metabolic intermediates not only supply energy but also shape immune phenotypes *via* signaling and epigenetic mechanisms.

In M1 polarization, stimuli like LPS stabilize HIF-1α and promote PKM2 tetramerization. Released ATP is converted to adenosine, activating A2aR to induce IL-10 (Toller-Kawahisa et al., 2025). Lactate exhibits a “double-edged sword” effect on the NLRP3 inflammasome. On one hand, lactate-mediated histone lactylation (e.g., H3K18) upregulates ALKBH5, reducing NLRP3 mRNA m6A modification and inhibiting inflammasome activation. On the other hand, in conditions like lupus pregnancy or SIRT3-deficient inflammation, lactate drives NLRP3 lactylation or metabolic shift toward glycolysis, promoting inflammasome activation and IL-1β secretion (Wei T. et al., 2021). Succinate from tumor-derived microvesicles triggers IDH2 and H3K122 succinylation, enhancing LDHA expression and glycolysis. Pim2 kinase directly phosphorylates glycolytic enzymes (PGK1, PDHA1, PFKFB2), driving M1 polarization in inflammatory arthritis.

M2 polarization is characterized by enhanced mitochondrial metabolism. Itaconate (*via* IRG1) inhibits TET2 to alleviate bone destruction and promotes vascular repair. Citrate redirects flux toward OXPHOS, supporting M2 polarization (Rong et al., 2025). Lactate can also drive M2 *via* RIG-I lactylation, inhibiting NF-κB. β-Hydroxybutyrate induces STAT1 β-hydroxybutyrylation, suppressing M1 gene expression. The aspartate-argininosuccinate shunt generates fumarate to activate antiviral RIG-I signaling (Bai et al., 2024).

In the tumor microenvironment, arginine-derived polyamines promote DNA hypomethylation in tumor-associated macrophages (TAMs), enhancing immunosuppression. Tryptophan metabolites (kynurenine, indole) activate AhR to drive resolution or TAM immunosuppression. The one-carbon enzyme MTHFD2 binds PTEN to modulate Akt and the M1/M2 balance (Shang et al., 2023). Drp1 interacts with HK1 to activate NLRP3 in periodontitis, while mitochondrial arginase-2 (Arg2) mediates IL-10-driven OXPHOS and resolution (Jiang et al., 2025).

Thus, macrophage metabolic reprogramming is a highly integrated global network encompassing glycolysis, FAO, amino acid/one-carbon metabolism, mitochondrial dynamics, and inflammatory signals. These metabolic circuits fine-tune epigenetic states, inflammasome activation, and cytokine expression, offering therapeutic targets for cancer, tissue repair, and immune diseases. Distinct metabolic programs and metabolite-mediated post-translational modifications that determine macrophage polarization phenotypes are depicted in Figure 3.

**FIGURE 3:**
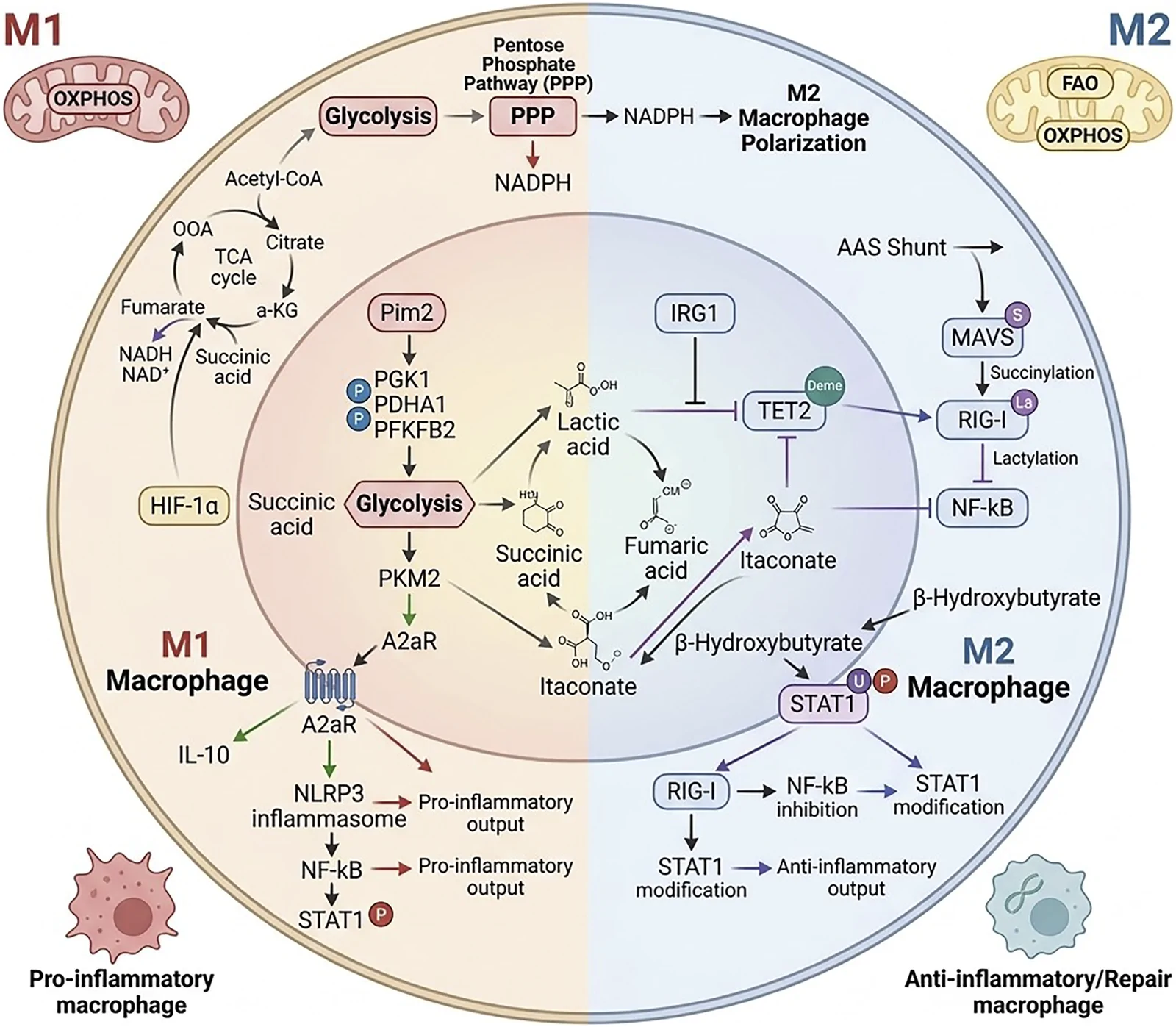
Metabolic reprogramming landscape during macrophage polarization. M1 macrophages rely on glycolysis and pentose phosphate pathway (PPP), whereas M2 macrophages prefer mitochondrial oxidative phosphorylation (OXPHOS) and fatty acid oxidation (FAO). Key metabolic intermediates (lactate, succinate, itaconate, β-hydroxybutyrate) trigger protein lactylation/succinylation, modulate HIF-1α, NLRP3 inflammasome and STAT1/NF-κB activity, and bidirectionally shape pro-inflammatory or tissue-repairing macrophage functional outputs.

### 2.3 The implications of macrophage polarization in rheumatoid arthritis

Macrophages play a critical role in the pathogenesis of RA, with their polarization status being closely linked to disease initiation and progression. Studies have demonstrated that aberrant macrophage polarization in the synovial tissue of RA patients contributes to persistent inflammation and joint destruction. Specifically, the activation state of synovial macrophages correlates significantly with disease activity and severity. Classically activated M1 macrophages, which are abundant in inflamed synovium, secrete high levels of pro-inflammatory cytokines and chemokines-such as TNF-α and IL-1β-that promote bone erosion and cartilage degradation, thereby exacerbating disease pathology (Kim et al., 2022).

In addition to the classic pathway, other inflammation-related signaling pathways together form a complex network for macrophage regulation. Animal studies further underscore the pivotal role of macrophages in RA. In a collagen-induced arthritis (CIA) mouse model, blockade of the macrophage scavenger receptor A (SR-A) markedly suppressed osteoclast differentiation and bone resorption. Treatment with an anti-SR-A neutralizing antibody significantly reduced the expression of osteoclast-related genes-including tartrate-resistant acid phosphatase (TRAP), cathepsin K (CTSK), and matrix metalloproteinase-9 (MMP-9)-in patient-derived cell cultures. *In vivo*, CIA mice exhibited attenuated bone erosion, along with decreased serum levels of C-terminal telopeptide and IL-6 (Yang et al., 2022). Elevated levels of IL-7 in RA synovial tissue and fluid promote the recruitment of monocytes into inflamed joints, where they differentiate into pro-inflammatory macrophages and mature osteoclasts, amplifying joint inflammation and destruction. Consequently, blockade of IL-7 or its receptor (IL-7R) signaling attenuates joint inflammation, osteoclastogenesis, and angiogenesis, highlighting the IL-7/IL-7R axis as a potential therapeutic target in RA (Meyer et al., 2022). Other studies have confirmed CCL2, CCL5, and CXCL10 promote osteoclastogenesis to a similar extent, with CCL5 exhibiting the strongest chemotactic effect on OCPs. These findings highlight several chemokine pathways as potential therapeutic targets in RA (Sucur et al., 2017).

Moreover, microRNAs (miRNAs) are increasingly recognized for their regulatory roles in macrophage polarization and RA progression. Multiple miRNAs have been implicated in modulating inflammatory responses and macrophage phenotype. For in-stance, altered miRNA expression profiles in peripheral blood mononuclear cells from RA patients correlate with disease activity. In experimental arthritis models, manipulation of specific miRNAs influences macrophage polarization and mitigates inflammatory responses, suggesting that miRNA-based strategies may offer novel therapeutic avenues for RA (Niu and Schulert, 2019).

Furthermore, cellular metabolic reprogramming has been proposed as an innovative therapeutic strategy to curb M1 macrophage expansion, modify the inflammatory milieu, and restore the balance between M1 and M2 macrophage populations (Zhao et al., 2017). Over the past decades, RA management has undergone substantial transformation, emphasizing the critical role of targeted therapies tailored to individual patients for improved disease control. Advances in understanding RA pathophysiology have guided the development of effective and safer treatments (Obeng et al., 2016). Accordingly, a growing number of biologic disease-modifying anti-rheumatic drugs (bDMARDs) have been developed and validated for clinical efficacy (Combe et al., 2015). By interfering with specific biological processes, bDMARDs initiated at very early stages can alter or even reverse RA progression (Yang et al., 2020).

Therefore, it is obvious that the polarization regulation of macrophages has a profound impact on the development process of rheumatoid arthritis, mainly including signal regulation, epigenetic regulation of pathways, and metabolic reprogramming. An increase in activated pro-inflammatory macrophages within the synovial tissue serves as an early hallmark of RA (Raimund et al., 2007). Consequently, early intervention following timely diagnosis is essential to prevent bone erosion, functional decline, and premature mortality in RA patients. Suppressing macrophage activation early represents an effective and well-tolerated therapeutic strategy for RA (Fu et al., 2023). Achieving remission within the “therapeutic window of opportunity” is associated with improved clinical outcomes; conversely, delayed treatment initiation correlates with prolonged symptom duration and unfavorable prognosis (Jacqueline et al., 2018). The identification of specific biochemical markers reflective of macrophage populations may provide valuable tools for detecting disease activity and represent potential therapeutic targets in RA, such as Tyrosine-protein kinase Mer(MerTK). Synovial tissues from RA patients in remission exhibit abundant MerTK^+^CD206^+^CD163^+^ M2 macrophages, whose presence inversely correlates with synovial thickening and vasculitis. Notably, such cells are also observed in healthy synovial tissues. In contrast, active RA is characterized by the prevalence of MerTK-CD206-macrophages within the synovial lining layer. This study further confirmed that patients with a high proportion of MerTK^+^CD206^+^ M2 macro-phages in the synovium are more likely to sustain remission, whereas those who relapse after discontinuation of biologic therapy show lower levels of these M2 macrophages (Alivernini et al., 2020). These findings suggest that MerTK^+^ macrophages exhibiting an M2 phenotype produce lipid mediators involved in inflammation resolution in both RA patients in remission and healthy subjects, and overexpress genes related to the regulation of local immune responses and tissue homeostasis. Induction of MerTK signaling has thus been proposed as a promising approach to promote remission in RA. Importantly, compelling evidence indicates a positive correlation between glucocorticoid treatment and enhanced MerTK expression on monocyte-derived macrophages, suggesting that this therapy may help alleviate RA-associated inflammation (Zizzo et al., 2012)

Unfortunately, there are currently no drugs specifically designed to modulate macrophage polarization in RA; most available therapies indirectly affect polarization by inhibiting the production of inflammatory cytokines such as TNF-α, IL-1β, and IL-6. Novel agents targeting these cytokines *via* monoclonal antibodies or soluble receptors may prove more effective during inflammatory stages of RA (Schatz et al., 2016). TNF inhibitors (e.g., infliximab, etanercept, adalimumab, golimumab, and certolizumab) bind both soluble and mem-brane-bound TNF-α, thereby inhibiting the activation of intracellular signaling path-ways that mediate pro-inflammatory effects. Tocilizumab inhibits IL-6-mediated inflammation by interacting with the IL-6 receptor, whereas anakinra a non-glycosylated recombinant form of the human IL-1 receptor antagonist-blocks the binding of IL-1β to its receptor, mitigating immunostimulatory and proinflammatory responses (Degboé et al., 2019).Notably, a recent study on the effects of anti-TNF agents on macrophages in RA patients revealed their ability to indirectly promote repolarization of pro-inflammatory M1 macrophages toward the M2 phenotype. Anti-TNF treatment not only suppressed inflammatory functions of macrophages *in vitro* but also enhanced their immunoregulatory functions, primarily *via* the IL-10/STAT3 signaling axis (Zou and Zhang, 2022). This shift in macrophage polarization within inflammatory contexts holds promise for improving the clinical efficacy of various bDMARDs in RA (Cao L. et al., 2022)

In summary, macrophage polarization played an important role in the pathogenesis of RA, and regulating it could effectively treat the symptoms of RA and delayed the progression of the RA. Therefore, traditional Chinese medicine has been given an important idea for treating RA. By regulating macrophage polarization, potential drugs and TCM formulas for treating RA can be screened.

## 3 Advances in the regulation of macrophage polarization by traditional Chinese medicine for the treatment of rheumatoid arthritis

TCM demonstrates considerable potential in the regulation of macrophage polarization. Accumulating evidence indicates that various TCM metabolites can modulate macrophage polarization, thereby exerting therapeutic effects across multiple diseases. Studies on TCM formulas have similarly revealed their capacity to influence macrophage polarization. For instance, Simiao San (SMS; Table 1) has been shown to ameliorate symptoms of acute gouty arthritis in mice by enhancing PI3K/Akt activation and promoting M2 macrophage polarization in joint tissues. *In vitro*, SMS treatment significantly suppressed monosodium urate (MSU)-induced inflammatory responses, increased the expression of p-Akt and Arg-1 in macrophages, and facilitated M2 polarization-effects that were abolished upon PI3K/Akt inhibition. Furthermore, SMS markedly reduced serum uric acid levels in hyperuricemia mice. Through mass spectrometry, the phytohormones and estrone were identified as potential active metabolites. These results suggest that SMS may exert anti-inflammatory effects by promoting M2 polarization, offering a promising therapeutic strategy for gouty arthritis and related conditions (Cao L. et al., 2022; Yang et al., 2021). The aqueous extract of ginseng and astragalus (AEGA) exhibits significant regulatory functions within the tumor microenvironment. *In vitro*, treatment of A549 cells with varying concentrations of AEGA significantly suppressed cell proliferation, while concurrently upregulating markers associated with M1 macrophages and downregulating markers of M2 tumor-associated macrophages (TAMs). *In vivo*, using a Lewis lung carcinoma (LLC) xenograft mouse model, administration of AEGA at different doses, alone or in combination with cisplatin (DDP), effectively inhibited tumor growth and ameliorated cisplatin-induced weight loss and immunosuppression. These findings collectively demonstrate that AEGA modulates macrophage polarization, thereby influencing cancer cell behavior and offering novel perspectives for comprehensive cancer therapy (Chen et al., 2019). The Xiaoshui Decoction (XSD) not only suppressed malignant pleural effusion formation, reduced pleural permeability and angiogenesis, and prolonged survival in a mouse model, but also promoted the repolarization of TAMs toward the M1 phenotype. *In vitro*, XSD markedly enhanced M1 macrophage expansion and reduced M2 macrophage populations through the induction of autophagy, suggesting its potential as a therapeutic agent for RA and other inflammatory conditions *via* modulation of macrophage polarization (Jin et al., 2020).These findings provide substantial evidence supporting the great potential of TCM in regulating macrophage polarization into its anti-inflammatory and immunomodulatory effects.

**TABLE 1:** TCM formulas regulate macrophage polarization in the treatment of RA.

Formula name	Source species	Experimental model	Target(s)	Mechanism of action	References
Wuwei Ganlu	*Rhododendron anthopogonoides* Maxim. [Ericaceae] *Juniperus angosturana* Ephedra sinica Stapf. [Ephedraceae] *Ephedra sinica Stapf* [Ephedraceae] *Myricaria platyphylla* Maxim. [Tamaricaceae] *Artemisia sieversiana* Ehrh. ex Willd. [Asteraceae]	*In Vitro* (THP-1 derived macrophages)	TNF-α, IL-6	Induces repolarization of M1 macrophages towards the M2 phenotype; inhibits secretion of pro-inflammatory cytokines TNF-α and IL-6	(Zheng et al., 2024)
Panax notoginseng polysaccharides	*Panax notoginseng* (Burkill) F.H.Chen [Araliaceae]	CIA rat model	JAK2-STAT3 signaling pathway	Inhibits JAK2/STAT3 pathway activation; promotes macrophage repolarization from pro-inflammatory M1 to anti-inflammatory M2 phenotype; scavenges reactive oxygen species (ROS); reduces levels of pro-inflammatory factors (TNF-α, IL-1β); increases expression of anti-inflammatory factor (TGF-β); alleviates joint swelling and bone erosion	(Yu et al., 2025)
Ganlu formula	*Rhododendron anthopogonoides* Maxim. [Ericaceae] *Juniperus angosturana Ephedra sinica* Stapf. [Ephedraceae] *Ephedra sinica Stapf* [Ephedraceae] *Myricaria platyphylla* Maxim. [Tamaricaceae] *Artemisia sieversiana* Ehrh. ex Willd. [Asteraceae]	CIA rat model, RAW264.7 cells	NLRP3, NF-κB, STAT3	Inhibits NLRP3 inflammasome activation; reduces M1 macrophage polarization; downregulates the NLRP3/Caspase-1/IL-1β signaling axis	(Zhang et al., 2024c)
Guizhi Shaoyao Zhimu Decoction	*Cinnamomum cassia* (L.) J. Presl [Lauraceae] *Cynanchum otophyllum* CK Schneid [Paeoniaceae] *Anemarrhena asphodeloides* Bunge [Liliaceae] *Glycyrrhiza uralensis* Fisch. ex DC.[Leguminosae] *Ephedra sinica* Stapf [Ephedraceae] *Zingiber officinale* Roscoe [Zingiberaceae] *Atractylodes macrocephala* Koidz [Asteraceae] *Saposhnikovia divaricata* (Turcz.) Schischk. [Umbelliferae] *Aconitum carmichaelii* Debeaux [Ranunculaceae]	CIA rat model	NF-κB signaling pathway	Inhibits NF-κB signaling transduction; reduces osteoclastogenesis; alleviates bone erosion	(Zhang et al., 2025)
Shanzhuyu-Baishao Combination	*Paeonia lactiflora* Pall. [Paeoniaceae] *Cornus officinalis* Siebold & Zucc. [Cornaceae]	CIA rat model	AMPK-mediated mitochondrial fission	Modulates mitochondrial fission *via* AMPK; promotes synovial cell apoptosis; attenuates inflammation	(Zhang et al., 2025)
Wuwei Ganlu (Variant)	*Rhododendron anthopogonoides* Maxim [Ericaceae] *Juniperus angosturana* R.P.Adams [Cupressaceae] *Ephedra sinica* Stapf [Ephedraceae] *Myricaria platyphylla* Maxim [Tamaricaceae]; *Artemisia sieversiana* Ehrh. ex Willd. [Asteraceae]	CIA rat model	JAK2, NF-κB, STAT3	Promotes macrophage polarization from pro-inflammatory M1 to anti-inflammatory M2 phenotype; reduces release of pro-inflammatory factors (TNF-α, IL-6); increases expression of anti-inflammatory factors (IL-10, Arg-1)	(Wen et al., 2024)
Wutou Decoction	*Aconitum carmichaelii* Debeaux [Ranunculaceae] *Astragalus mongholicus* Bunge [Fabaceae] *Glycyrrhiza uralensis* Fisch. ex DC. [Fabaceae] *Ephedra sinica* Stapf [Ephedraceae] *Paeonia lactiflora* Pall. [Paeoniaceae]	CIA rat model, RAW264.7 cells	NF-κB, PPARγ, M1/M2 macrophage polarization	Inhibits NF-κB path (Gao and Guo, 2024)way reducing pro-inflammatory cytokine secretion; promotes PPARγ nuclear translocation enhancing anti-inflammatory effects; modulates macrophage polarization	(Lin et al., 2023a)
Duhuo Jisheng Decoction	*Angelica dahurica* (Hoffm.) Benth. and Hook.f. [Apiaceae] *Taxillus chinensis* (DC.) Danser [Loranthaceae] *Eucommia ulmoides* Oliv. [Eucommiaceae] *Achyranthes bidentata* Blume [Amaranthaceae]; *Asarum heterotropoides* F.Schmidt [Aristolochiaceae] *Saposhnikovia divaricata* (Turcz. ex Ledeb.) Schischk. [Apiaceae] *Gentiana macrophylla* Pall. [Gentianaceae] *Neolitsea cassia* (L.) Kosterm. [Lauraceae] *Angelica sinensis* (Oliv.) Diels [Apiaceae] *Conioselinum anthriscoides* ‘Chuanxiong’ [Apiaceae] *Cornus officinalis* Siebold & Zucc. [Cornaceae]; *Rehmannia glutinosa* (Gaertn.) Libosch. ex DC. [Orobanchaceae] *Panax ginseng* C.A.Mey. [Araliaceae] *Smilax glabra* Roxb. [Smilacaceae] *Glycyrrhiza glabra* L. [Fabaceae]	TNF-α transgenic (TNF-Tg) mouse model	M1 macrophages, Lymphangiogenesis	Reduces M1 macrophage infiltration; promotes lymphangiogenesis and drainage function; alleviates joint inflammation	(Gao and Guo, 2024; Zheng et al., 2022)
Simiao San	*Atractylodes lancea* (Thunb.) DC. [Asteraceae]; *Phellodendron amurense* Rupr. [Rutaceae]; *Achyranthes bidentata* Blume [Amaranthaceae] *Coix lacryma-jobi* L. [Poaceae]	Rat bone marrow-derived macrophages	M1 macrophages (CD68, iNOS)	Inhibits LPS + IFN-γ-induced M1 polarization; shows no significant effect on IL-4+IL-13-induced M2 polarization	(Cao et al., 2022a; Yang et al., 2021)
Xinfeng Capsule	*Astragalus mongholicus* Bunge [Fabaceae] *Coix lacryma-jobi* L. [Poaceae] *Tripterygium wilfordii* Hook.f. [Celastraceae] *Scolopendra subspinipes* mutilans L. Koch [Scolopendridae]	Clinical study in RA patients	CD86, CD206, RF, ESR	Decreases M1 macrophage marker CD86; increases M2 macrophage marker CD206; improves inflammatory and immune markers	(Zheng et al., 2022; Zhao et al., 2023)
Jinteng Qingbi Granules	*Lonicera japonica* Thunb. [Caprifoliaceae] *Cornus officinalis* Siebold & Zucc. [Cornaceae] *Sinomenium acutum* (Thunb.) Rehder and E.H.Wilson [Menispermaceae] *Scleromitrion diffusum* (Willd.) R.J.Wang [Rubiaceae] *Scrophularia ningpoensis* Hemsl. [Scrophulariaceae]; *Rehmannia glutinosa* (Gaertn.) Libosch. ex DC. [Orobanchaceae]	CIA rat model	TLR4/NF-κB, Th17/Treg	Modulates M1/M2 macrophage polarization; balances Th17/Treg ratio; inhibits TLR4/NF-κB pathway	(Tang et al., 2022)
Huangqi Guizhi Wuwu Decoction	*Astragalus mongholicus* Bunge [Fabaceae] *Neolitsea cassia* (L.) Kosterm. [Lauraceae] *Cornus officinalis* Siebold & Zucc. [Cornaceae] *Zingiber officinale* Roscoe [Zingiberaceae] *Ziziphus jujuba* Mill. [Rhamnaceae]	Arthritis rat model	α7nAChR receptor	Promotes conversion from M1 to M2 phenotype; elevates levels of anti-inflammatory factors IL-10 and TGF-β; ameliorates inflammatory response	(Zheng et al., 2022)
Sanbaibang(feilongzhangxue)	*Zanthoxylum asiaticum* (L.) Appelhans, Groppo and J.Wen [Rutaceae]	CIA rat model	TLR4/NF-κB signaling pathway	Inhibits inflammatory response; promotes macrophage polarization towards M2 phenotype; alleviates arthritis symptoms	(Zhan et al., 2023)
Tianshan Snow Lotus Oral Liquid	*Saussurea involucrata* (Kar. and Kir.) Sch.Bip. [Asteraceae]	CIA rat model	Notch1 signaling pathway, Macrophage polarization (M1/M2)	Inhibits Notch1 signaling pathway; promotes macrophage polarization towards M2 phenotype; reduces secretion of pro-inflammatory factors (TNF-α, IL-1β, IL-6)	(Saimire et al., 2025)
Wenhua Juanbi Formula	*Hansenia weberbaueriana* (Fedde ex H.Wolff) Pimenov & Kljuykov [Apiaceae] *Angelica dahurica* (Hoffm.) Benth. and Hook.f. [Apiaceae] *Saposhnikovia divaricata* (Turcz. ex Ledeb.) Schischk. [Apiaceae] *Gentiana macrophylla* Pall. [Gentianaceae] *Neolitsea cassia* (L.) Kosterm. [Lauraceae] *Angelica sinensis* (Oliv.) Diels [Apiaceae] *Cornus officinalis* Siebold & Zucc. [Cornaceae] *Glycyrrhiza glabra* L. [Fabaceae]	CIA rat model	Autophagy-related proteins (Beclin1, LC3 II), Macrophage polarization (CD16/CD206)	Inhibits autophagy; reduces M1 macrophage polarization; increases M2 polarization; decreases IL-6, TNF-α; increases IL-10, Arginase1	(Zhou and Liu, 2025; Liu and Gao, 2022)
Yiqi Yangyin Tongluo Formula	*Adenophora triphylla* (Thunb.) A.DC. [Campanulaceae] *Glehnia littoralis* (J.G.Cooper) F.Schmidt ex Miq. [Apiaceae] *Ophiopogon japonicus* (Thunb.) Ker Gawl. [Asparagaceae] *Schisandra chinensis* (Turcz.) Baill. [Schisandraceae] *Neolitsea cassia* (L.) Kosterm. [Lauraceae] *Rehmannia glutinosa* (Gaertn.) Libosch. ex DC. [Orobanchaceae] *Salvia miltiorrhiza* Bunge [Lamiaceae] *Conioselinum anthriscoides* ‘Chuanxiong’ [Apiaceae] *Leonurus japonicus* Houtt. [Lamiaceae]	RAW264.7 macrophage cell line	CASP3, BAX, BCL2, PTGS2	Promotes macrophage apoptosis; inhibits inflammatory response; modulates TNF, IL-17, and P53 signaling pathways	(Lin et al., 2025)

### 3.1 TCM formulas in the treatment of RA *via* macrophage regulation

At present, the research on TCM formulas mainly focuses on the level of signaling pathways. Table 1 provides a summary of the mechanisms by which various TCM formulas modulate macrophage polarization in the treatment of RA. Liuwei Dihuang Wan ameliorated disease progression by modulating methylation levels of the DNMT1 and ER-α genes. The Panax notoginseng polysaccharide–deoxycholic acid–folic acid composite micelle promoted M2 macrophage polarization and alleviated joint inflammation in a CIA rat model through suppression of the JAK2/STAT3 signaling pathway. Similarly, Wuwei Ganlu (Table 1), tested in a THP-1-derived macrophage model, inhibited the secretion of pro-inflammatory cytokines TNF-α and IL-6 and facilitated the transition from M1 to M2 macrophage phenotypes. Ganlu formula demonstrated efficacy in both CIA rats and RAW264.7 cells by suppressing NLRP3 inflammasome activation, thereby reducing M1 polarization. Furthermore, Guizhi Shaoyao Zhimu Decoction (Table 1) attenuated bone erosion in a collagen-induced arthritis model *via* inhibition of the NF-κB signaling pathway. In another study, Qizhen Buxue Decoction (QBD) was found to regulate macrophage polarization and inflammation *via* multiple signaling pathways, as validated through network pharmacology and experimental approaches. Active metabolites of QBD and their corresponding targets were initially identified from databases, alongside targets associated with macrophage polarization and inflammation. Integrated network analyses were constructed to pinpoint key metabolites, primary biological processes, and signaling pathways. Molecular docking assays confirmed the binding affinity between most candidate metabolites and their targets. *In vitro*, serum from QBD-treated mice upregulated the expression of the α7 nicotinic acetylcholine receptor (α7 nAChR), reduced M1 macrophage abundance, stimulated M2 macrophage generation, suppressed pro-inflammatory cytokines (IL-6 and IL-1β), and enhanced anti-inflammatory cytokines (IL-10 and TGF-β1), collectively modulating macrophage polarization and inflammatory responses (Wang S. et al., 2023).

Collectively, these studies underscore the significant role of TCM formulas in modulating macrophage polarization through diverse signaling pathways, highlighting their potential as therapeutic agents for RA (Table 1). At present, research on the pathological aspects of RA mainly focuses on synovitis and bone destruction, both of which are caused by the release of polarized inflammatory factors by macrophages and changes in the immune environment inside the joint cavity. The current research uses the CIA rat model to simulate pathological changes in the body, focusing on TCM Formulas intervention in inflammatory signaling pathways, such as JAK-STAT signaling, NF-κB signaling, NLRP3 inflammasome activation. These formulas promoted macrophage polarization from pro-inflammatory M1 to anti-inflammatory M2 phenotype and reduced release of pro-inflammatory factors (TNF-α, IL-6), increased expression of anti-inflammatory factors (IL-10, Arg-1). At present, the research on the treatment mechanism of these TMC formulas was not deep enough. Firstly, due to the complex chemical composition of TCM formulas, although research tools such as LC-MS and network pharmacology have been developed, their accuracy is still insufficient, Secondly, the vast majority of disease models still use the CIA rat model, and advanced disease research models such as cell experiments and gene knockout animals are rarely used, Restricted in-depth research on the mechanism of TCM formulas regulating macrophage polarization in the treatment of RA. The use of new research methods is highly necessary in TCM metabolite-based therapy for regulating macrophage polarization in the treatment of RA (Table 1).

### 3.2 Regulating the active metabolites of traditional Chinese medicine for treating RA through macrophages

As research progresses, an increasing number of TCM active metabolites with macrophage polarization-modulating properties have been identified. Compared with the study of the treatment mechanism of TCM formulas, the research content of TCM metabolites is more abundant and in-depth, in addition to regulating common inflammations such as NF- κB signaling and NLRP3 inflammasome activation. There have been indepth studies on the regulation of upstream Th17/Treg balance, PI3K/Akt/SGK/FoxO signaling pathway, TLR-MyD88/IRAK4-IRF5 signaling axis, as well as the effects of Autophagy and glucose metabolism on macrophages. Table 2 summarizes the mechanisms by which various metabolites derived from TCM modulate macrophage polarization in the treatment of RA. For example, icariin (ICA) suppresses macrophage glycolysis *via* the ERK/HIF-1α/GLUT1 pathway in a CIA rat model, thereby facilitating the transition from M1 to M2 polarization. Polyphyllin I (PPI) dually regulates macrophage polarization through the JAK2-STAT3 signaling pathway in both LPS-stimulated RAW264.7 cells and a CIA rat model. Ginsenoside Rh2 targets the NLRP3/caspase-11/GSDMD-N pathway in CIA rats, concurrently inhibiting macrophage pyroptosis and promoting M2 polarization. Punicalagin exerts anti-inflammatory effects in a CIA mouse model by modulating the NF-κB signaling pathway and suppressing NLRP3 inflammasome activation. Gastrodin was found to upregulate the mRNA expression of M2 macrophage markers Mgl2 and Mrc1 in murine RAW264.7 macrophages, while concurrently enhancing arginase activity. These effects were abolished upon treatment with BCL6 siRNA, indicating that gastrodin promotes M2-like macrophage polarization through the induction of BCL6 expression. Consequently, this shift confers protection against oxidative stress-induced apoptosis and cell death in macrophages (Jia et al., 2017). Similarly, icariin has been demonstrated to suppress M1 polarization and promote M2 polarization, thereby reducing the release of inflammatory cytokines and inhibiting the progression of osteoarthritis. These findings not only provide novel therapeutic insights for inflammatory conditions such as osteoarthritis but also underscore the considerable developmental value of TCM-derived metabolites in macrophage polarization research and related disease treatment (Wang FF. et al., 2023).

**TABLE 2:** TCM metabolites for treating rheumatoid arthritis by regulating macrophage polarization through inflammation related pathways.

No.	Metabolites	Source species	Assay type	Experimental model	Dose range	Controls	Experimental duration	Mechanism of action	References
1	Punicalagin	*Punica granatum* L.[Lythraceae]	*In Vitro*	Bone marrow macrophages (BMMs)	Various concentrations (up to 200 μmol/L)	Untreated control	0, 12, 24, 48 h	Inhibits M1 macrophage polarization; promotes M2 macrophage conversion; inhibits macrophage pyroptosis	(Gao et al., 2025)
				BMMs stimulated with LPS (100 ng/mL) and IFN-γ (20 ng/mL)	20 μmol/L (low), 50 μmol/L (high)	Control (untreated), LPS/IFN-γ only	12 h		
				BMMs stimulated with LPS (100 ng/mL) and ATP (5 mmol/L)	20 μmol/L (low), 50 μmol/L (high)	Control (untreated), LPS/ATP only	12–16 h		
				RAW264.7 cells and BMMs stimulated with LPS/IFN-γ or LPS/ATP	50 μmol/L	Control (untreated), LPS/IFN-γ or LPS/ATP only	30 min to 12 h		
			*In Vivo*	Collagen-Induced Arthritis (CIA) DBA/1 mice	10 mg/kg (low), 20 mg/kg (high)	Control (PBS), CIA model (PBS)	Day 22–49 (27 days treatment)		
				CIA DBA/1 mice	10 mg/kg (low), 20 mg/kg (high)	Control (PBS), CIA model (PBS)	Day 22–49		
				CIA mouse synovial tissue	10 mg/kg (low), 20 mg/kg (high)	Control (PBS), CIA model (PBS)	Day 22–49		
				CIA mouse synovial tissue	10 mg/kg (low), 20 mg/kg (high)	Control (PBS), CIA model (PBS)	Day 22–49		
				CIA mouse synovial tissue	10 mg/kg (low), 20 mg/kg (high)	Control (PBS), CIA model (PBS)	Day 22–49		
2	Aristolochic acid II(AAIVa)	*Aristolochia debilis* Siebold & Zucc.[Aristolochiaceae]	*In Vitro*	RAW 264.7 murine macrophage cell line	50 μM, 100 μM (based on previous study); 100 μM used for main experiments	Untreated cells (negative control), LPS-stimulated cells (disease/positive control)	12 h	Promotes M2 macrophage polarization; modulates Th17/Treg ratio; reduces levels of IL-6, IL-1β, TNF-α	(Li et al., 2022)
			*In Vivo*	SKG mice (BALB/c background, male, 6–8 weeks)	3 mg/kg, 10 mg/kg	Vehicle control (solvent), Positive control: Methotrexate (2 mg/kg)	4 weeks of treatment (from day 12 to day 42 post-arthritis induction)		
				Bone Marrow-Derived Macrophages (BMDMs) from SKG and BALB/c mice	100 μM	Untreated cells, LPS-stimulated cells (M1 inducer)	24 h (post-LPS stimulation)		
				Spleen primary cells from SKG and BALB/c mice	100 μM	Untreated cells, LPS-stimulated cells (inflammatory stimulus)	Not explicitly stated, standard culture period for cell differentiation		
3	Wilforlide A	*Tripterygium wilfordii* Hook. f.[Celastraceae]	*In Vitro*	THP-1 derived macrophages	1, 5, 10, 20, 40, 80, 160, 300 ng/mL	Untreated cells (negative control)	48 h	suppressed LPS/IFN-γ-induced TLR4 upregulation, IκBα degradation and NF-κB p65 activation	(Wu et al., 2023)
				THP-1 derived macrophages stimulated with LPS/IFN-γ	5 ng/mL	Untreated cells, LPS/IFN-γ-stimulated cells (disease model), TAK242 (TLR4 inhibitor, 10 ng/mL) as a mechanistic control	Pre-treatment for 3 h, followed by LPS/IFN-γ stimulation for 30 min (signaling) or 48 h (polarization)		
			*In Vivo*	Collagen-Induced Arthritis (CIA) DBA/1 male mice	30 mg/kg, 60 mg/kg (i.p.)	Vehicle control (10% DMSO in saline), Disease model (CIA), No positive control drug mentioned	Treatment from day 15 to day 42 after the first immunization (27 days total)		
4	Astragalin	*Astragalus membranaceus* Bunge[Fabaceae]	*In Vitro*	THP-1 derived human macrophages	1–5 ng/mL	Negative: Untreated cells	3h pre-treatment + 48h stimulation	Reduces pro-inflammatory cytokine release; promotes polarization from M1 to M2	(Gao and Guo, 2024)
				LPS/IFN-γ induced M1 polarization		Positive: TAK242 (TLR4 inhibitor)			
				​		Disease: LPS/IFN-γ stimulation			
			*In Vivo*	DBA/1 mice	30–60 mg/kg	Vehicle control (10% DMSO)	27 days treatment		
				Collagen-Induced Arthritis (CIA)		Disease: CIA model	(Day 15–42 post-immunization)		
5	Geniposide	*Gardenia jasminoides* J. Ellis[Rubiaceae]	*In Vitro*	Bone Marrow-Derived Macrophages (BMDMs) from CIA mice	Not specified (*ex vivo* analysis)	Normal, CIA, GE-treated	Not specified	inhibition of SphK1 membrane targeting, restoring the polarization balance of macrophages	(Zhang et al., 2023)
				RAW264.7 macrophage cell line (M1 polarization: LPS 100 ng/mL + IFN-γ 20 ng/mL)	25, 50, 100 µM	Control (M0), M1 (LPS + IFN-γ), M2 (IL-4), PF543 (100 nM), FTY720 (5 µM)	24 h		
				RAW264.7 cells (SphK1 kinase activity assay)	0.032–500 µM	PF543 (IC_50_ = 629.2 nM)	2 h (kinase reaction)		
			*In Vivo*	Collagen-Induced Arthritis (CIA) mice (DBA/1 male, 7–8 weeks)	60 and 120 mg/kg/day	Normal, CIA, MTX (1.0 mg/kg/3 days)	14 days (d28–d41)		
6	Theaflavin-3,3′-digallate (TFDG)	*Camellia sinensis* (L.) Kuntze[Theaceae]	*In Vitro*	Bone Marrow Macrophages (BMMs) from DBA/1 mice	0.1 and 1.0 μmol/L	PBS, TNF-α (10 ng/mL)	24 h (after 2h pretreatment)	attenuating the inflammation and promoting anti-inflammatory M2 macrophage polarization through controlling autophagy	(Yang et al., 2024)
				BMMs (cytotoxicity assay)	0.01–100 μmol/L	Untreated cells (0 μmol/L)	1–7 days		
				BMMs (autophagy and polarization with inhibitor)	1.0 μmol/L TFDG + 3-MA (autophagy inhibitor)	TNF-α, TFDG alone, 3-MA control	24 h		
			*In Vivo*	Collagen-Induced Arthritis (CIA) DBA/1 mice (8–10 weeks, male)	1 and 10 mg/kg/day	Sham (no CIA), Vehicle (CIA + saline)	8 weeks (6 days/week)		
7	Koumine Hydrochloride	*Gelsemium elegans* (Gardner & Champ.) Benth.[Gelsemiaceae]	*In Vitro*	RAW264.7 Cells (M1 polarization)	Not specified (concentration used: 200 µM)	LPS (Inducer), Untreated (Negative)	24 h	inhibited M1 macrophage polarization and had an inhibitory effect on the PI3K/AKT signaling pathway	​
				RAW264.7 Cells (M2 polarization)	Not specified (concentration used: 200 µM)	IL-4 (Inducer), Untreated (Negative)	24 h		
				RAW264.7 Cells (Mixed polarization)	Not specified (concentration used: 200 µM)	LPS + IL-4 (Inducer), Untreated (Negative)	24 h		
			*In Vivo*	AIA Lewis Rats	0.6, 3, 15 mg/kg/day	NS (Negative), MTX (Positive)	10 days (D14-D23)		
				CIA C57BL/6 J Mice	10 mg/kg/day	NS (Negative)	10 days (D35-D44)		
8	paeoniforin-6′-0-benzene sulfonate(CP-25)	*Paeonia lactiflora* Pall.[Ranunculaceae]	*In Vitro*	Raw264.7 macrophages	PGE2: 10 μMol/L; CP-25: 1 μMol/L; Paroxetine: 2.5 μMol/L	Untreated, PGE2, Paroxetine (positive control)	Not specified (transient transfection/treatment)	regulates the functions of B cells through BAFF-TRAF2-NF-κB signaling	(Chen and Jiao, 2024)
				HEK293 cells (FRET, Co-IP, luciferase)	Plasmid transfection; CP-25: 1 μMol/L	Empty vector, mutant plasmids	24–48 h		
				HUVEC tube formation assay	CP-25: 0.1–10 μMol/L	Untreated, VEGF-stimulated	Not specified (typically 6–18 h)		
				Human RA synovial macrophages	GSK180736A (GRK2 inhibitor); CP-25: 1 μMol/L	Untreated, vehicle	Not specified (short-term)		
9	ephedrine (EPH)	*Ephedra saxatilis* (Stapf) Royle ex Florin[Ephedraceae]	*In Vitro*	MH7A cells (human RA synovial fibroblasts)	1–6 mg/mL	Control (normal culture), TNF-Î± group, TNF-Î±+EPH group	24–48 h	inhibit M1 macrophage polarization, and reduce the expressions of TLR8, MyD88, and TRAF6 proteins in cartilage tissue	(Wei et al., 2021b)
			​	THP-1-derived macrophages	6 mg/mL	M0, M1, M1+EPH, vector + M1, TLR8+M1, TLR8+EPH + M1, siNC, siTLR8	36–48 h		
			*In Vivo*	Wistar rats (FCA-induced RA model)	20–40 mg/kg	Sham, Model, EPH-L, EPH-H	21 days		
10	Lonicerin (LON)	*Lonicera japonica* Thunb.[Caprifoliaceae]	*In Vitro*	RAW264.7 macrophages	3–30 μMol/L	Control (medium), LPS/IFN-γ (M1 induction), IL-4 (M2 induction), shCtrl (control shRNA), shP65 (P65 knockdown)	24 h	inhibited NLRP3 inflammasome activation in M1 macrophages, thereby reducing inflammation by inhibiting IL-1β and IL-18 release	(Gao and Guo, 2024)
			​	Bone Marrow-Derived Macrophages (BMDM)	3–30 μMol/L	Control, LPS/IFN-γ, IL-4	24 h		
			​	293T cells (for lentivirus production)	N/A	shCtrl, shP65	48–72 h		
			*In Vivo*	DBA/1 mice (CIA model)	3–30 mg/kg	Normal control, CIA model, Dexamethasone (DXM, 1 mg/kg) as positive control	23 days (from day 22 to day 44 post-immunization)		
11	sinomenine	*Sinomenium acutum* (Thunb.) Rehder and E.H.Wilson.[Menispermaceae]	*In Vitro*	THP-1 human monocytic leukemia cell line (differentiated to macrophages)	10–200 μMol/L	Normal control (untreated), Model group (LPS 1 μg/mL + IFN-γ 20 ng/mL)	Pre-incubation: 12 h; LPS/IFN-γ stimulation: 4 h; Total: 16 h	Upregulation of the expression level of paired immunoglobulin like receptor B (PIR-B) and inhibition of classical activation of macrophages	​
			​	293T cells (for lentivirus production)	N/A	shCtrl, shP65	48–72 h	​	​
			*In Vivo*	DBA/1 mice (CIA model)	3–30 mg/kg	Normal control, CIA model, Dexamethasone (DXM, 1 mg/kg) as positive control	23 days (from day 22 to day 44 post-immunization)		

#### 3.2.1 TCM metabolites regulate macrophage polarization through inflammation related signaling pathways of RA

Multiple active metabolites from Chinese medicines target the core inflammatory signaling axes to reshape the M1/M2 balance of macrophages, representing the most thoroughly investigated mechanistic direction in current research. Metabolites such as ellagic acid, kaempferol, lignans, ephedrine, and lonicerin inhibit the TLR4/NF-κB, NLRP3 inflammasome, JAK2/STAT3, and PI3K/AKT pathways, leading to downregulation of M1 markers including iNOS, TNF-α, and IL-1β. Brucine, sinomenine, and aristolochic acid IIA upregulate the immunoinhibitory receptor PIR-B while concurrently restoring the Th17/Treg balance. Triptophenolide and the paeoniflorin derivative CP-25 block macrophage pyroptosis and reduce synovial inflammatory infiltration. Theaflavin and geniposide promote M2-type polarization through autophagy-related pathways, with elevated expression of Arg-1 and IL-10. Both *In Vitro* cellular experiments (RAW264.7, THP-1, and primary bone marrow-derived macrophages) and animal models (CIA, SKG, and AIA) have validated that these metabolites alleviate joint swelling and inhibit osteoclastic differentiation and cartilage erosion (Table 2).

#### 3.2.2 TCM metabolites regulate macrophage polarization through epigenetic regulation of RA

Chinese medicine metabolites stabilize the reparative macrophage phenotype through a three-tiered epigenetic regulatory network involving DNA methylation, histone modifications, and non-coding RNAs. α-Mangostin and curcumin synergistically activate the SIRT1/PPARγ signaling axis, modulating histone acetylation and suppressing the transcription of pro-inflammatory genes. Pharmacological intervention upregulates TET family demethylases and downregulates DNMT3A, thereby relieving the methylation-mediated silencing of key M2-associated transcription factors such as KLF4 and PPARγ. Concurrently, these agents modulate lncRNAs and miRNAs (e.g., miR-34a) that target KLF4 and STAT6, inhibiting the expression of M1-polarization-related genes. Epigenetic regulation sustains long-term macrophage immune homeostasis, and several metabolites have undergone small-sample clinical validation in RA patients, demonstrating protracted anti-inflammatory advantages(Table 3).

**TABLE 3:** TCM metabolites for treating rheumatoid arthritis through epigenetic regulation of macrophage polarization.

No.	Metabolites	Source species	Parameter	Experimental model	Dose range	Controls	Experimental duration	Mechanism of action	References
1	α-Mangostin (MG)	*Garcinia mangostana* L.[Clusiaceae]	*In Vivo*	AIA mouse model (Antigen-Induced Arthritis)	MG: 58 mg/kg (single dose) NAM: 72 mg/kg T007097: 15 mg/kg	Normal control (healthy mice) AIA model (0.5% CMC-Na vehicle) MG + NAM MG + T007097	28 days total Treatment from day 21–28 (once daily)	Synchronously activating SIRT1 and PPAR-γ was revealed to be necessary by MG to repress inflammatory responses in THP-1 monocytes	(Gan et al., 2022)
				Mouse spleen macrophages (*ex vivo* analysis)	N/A (derived from treated mice)	Same as *in vivo* groups	Analysis at endpoint (day 28)		
				Mouse joint tissue (immunofluorescence)	N/A (tissue from treated mice)	Same as *in vivo* groups	Analysis at endpoint (day 28)		
			*In Vitro*	THP-1 human monocytes	MG: 5 μg/mL Sirtinol: 30 μM T007097: 10 μM LPS: 2 μg/mL	Normal cells LPS-stimulated cells MG + sirtinol MG + T007097 siRNA controls	LPS stimulation: 8 h Treatment: 24 h Transfection: 48 h		
				THP-1 cells (siRNA transfection)	si-SIRT1, si-PPAR-γ transfection	Negative control siRNA Gene-specific siRNA	Transfection: 48 h		
				Molecular docking	PPAR-γ protein (PDB ID: 4YT1)	N/A	N/A		
2	Curcumin	*Curcuma longa* L.[Zingiberaceae]	*In Vitro*	RAW264.7 macrophages	Not specified (various studies)	LPS/IFN-γ stimulated cells; Untreated cells	Varies by study (e.g., pre-treatment before stimulation)	Binds PPAR-γ and activates its signaling pathway; upregulates SIRT1 expression; inhibits M1 polarization; reduces pro-inflammatory cytokines (TNF-α, IL-1β)	(Lu et al., 2023)
				THP-1 monocytes (human)	Not specified	LPS-stimulated cells	Not specified		
				Fibroblast-like synoviocytes (FLSs) from RA patients	Curcumin pre-treatment (e.g., before TNF-α stimulation)	TNF-α stimulated FLSs; Untreated FLSs	Not specified		
				Microglia (CNS macrophages)	Not specified	LPS-stimulated cells	Not specified		
			*In Vivo*	Collagen-Induced Arthritis (CIA) rats	Not specified (various dosing regimens)	CIA model control; Vehicle control	Varies by study (e.g., throughout arthritis development)		
				Adjuvant-Induced Arthritis (AIA) rats/mice	Curcumin alone or with Methotrexate	AIA model control; Vehicle control; Methotrexate group	Treatment during established arthritis		
				K/BxN serum-transfer arthritis (STA) mice	N/A (model characterization)	Healthy controls; STA model	Observation at inflammation onset		
				Clinical Trials (Human RA Patients)	500 mg/day (Chandran and Goel, 2012); 250 mg/day (Novel formulation, Amalraj et al., 2017); CuroWhite™ (Jacob et al., 2019)	Placebo; Diclofenac sodium (active comparator)	8 weeks to 3 months		
				Human RA Patients (Metabolic Study)	Curcumin supplementation	Placebo	Intervention period (specific duration not detailed in summary)		
				THP-1 derived macrophages (RNA-seq and metabolism)	5 μg/mL	DMSO, LPS + IFN-γ, 2-DG (8.2 mg/mL)	6 h (RNA-seq) or 24 h (component measurement)		
			*In Vivo*	Collagen-Induced Arthritis (CIA) mice (DBA1/J male, 8–10 weeks)	5 mg/kg	Vehicle (PBS), Positive control: MTX (1.5 mg/kg)	18 days (from day 20 to day 38)		

#### 3.2.3 TCM metabolites regulate macrophage polarization through metabolic reprogramming of RA

Macrophage glucose/lipid metabolism and mitochondrial function serve as core drivers dictating polarization phenotypes, and Chinese medicine metabolites can target metabolic pathways to reverse the M1-skewed metabolic profile in the RA synovium. Icariin and ginsenoside Rh2 block the HIF-1α/GLUT1 glycolytic pathway, reduce lactate production, and curb excessive M1 activation. Artemisinin and cepharanthine downregulate key glycolytic enzymes while promoting mitochondrial oxidative phosphorylation (OXPHOS) and fatty acid oxidation (FAO). Acanthoside B inhibits PDE4, thereby disrupting the inflammatory crosstalk between macrophages and fibroblast-like synoviocytes (FLS). Metabolic intermediates such as itaconate and β-hydroxybutyrate mediate post-translational modifications of STAT1 and NF-κB, bidirectionally modulating inflammation and tissue repair. Nano-delivery systems improve aqueous solubility and enable targeted delivery to synovial macrophages, amplifying the metabolic reprogramming effects and substantially enhancing *in vivo* anti-arthritic efficacy(Table 4).

**TABLE 4:** TCM metabolites for treating rheumatoid arthritis by regulating macrophage polarization through Metabolic reprogramming.

No.	Metabolites	Source species	Parameter	Experimental model	Dose range	Controls	Experimental duration	Mechanism of action	References
1	Icariin(ICA)	*Epimedium rotundatum* K.S.Hao.[Berberidaceae]	*In Vitro*	RAW264.7 macrophage cell line induced with LPS (100 ng/mL) + IFN-γ (20 ng/mL)	ICA: 40 μg/mL ADSCs-EXO: 40 μg/mL ADSCs-EXO-ICA: 80 μg/mL (containing 40 μg/mL ICA + 40 μg/mL ADSCs-EXO)	Blank (untreated cells) LPS + IFN-γ (inflammatory model) ICA, ADSCs-EXO, ADSCs-EXO-ICA treatment groups	M1 induction: 24 h Drug treatment: 48 h	Arrests RA fibroblast-like synoviocytes (RAFLS) at G2 phase by inhibiting cyclin B1; promotes macrophage repolarization from M1 to M2 phenotype	(Ge et al., 2022)
			*In Vivo*	Collagen-Induced Arthritis (CIA) rat model	ICA: 100 μg/rat ADSCs-EXO: 100 μg/rat ADSCs-EXO-ICA200: 200 μg/rat ADSCs-EXO-ICA400: 400 μg/rat MTX: 1 mg/kg	Control (healthy rats) CIA (disease model) ICA, ADSCs-EXO, ADSCs-EXO-ICA200/400, MTX treatment groups (MTX as positive control)	Treatment: 8 injections every other day (16 days total) Total study duration: 28 days		
2	Ginsenoside Rh2	*Panax ginseng* C. A. Mey.[Araliaceae]	*In Vitro*	RAW264.7 mouse macrophage cell line	Rh2: 5–80 µM	LPS(−) negative control	24 h	Inhibits macrophage glycolysis; promotes macrophage polarization from M1 to M2 phenotype	(Con et al., 2025)
					MSC-exo: 1–10 μg/mL	LPS(+) and PBS vehicle control			
						LPS(+) and MTX positive control			
				RAW264.7 cells and L929 fibroblasts	MSC-exo: 20 μg/mL	Non-LPS treated cells	4 h		
						L929 fibroblast control			
				RAW264.7 cells	Rh2: 5–80 µM	LPS(−) negative control	24 h		
					MSC-exo: 1–10 μg/mL	LPS(+) and PBS vehicle control			
​	​	​	*In Vivo*	CIA rat model	MSC-exo: 500 μg/kg	PBS control group	4 h post-injection	​	​
					Rh2: 5 mg/kg				
				Collagen-Induced Arthritis (CIA) rat model	MSC-exo: 500 μg/kg	Normal control	52 days total		
					Rh2: 5 mg/kg	Model control (PBS)	28 days treatment		
					MTX: 5 mg/kg	MTX positive control			
				CIA rat ankle joints	MSC-exo: 500 μg/kg	Normal control	52 days		
					Rh2: 5 mg/kg	Model control (PBS)			
						MTX positive control			
				CIA rat knee joints	MSC-exo: 500 μg/kg	Normal control	52 days		
					Rh2: 5 mg/kg	Model control			
3	Syringin	*Eleutherococcus senticosus*(Rupr.&Maxim.)Maxim[Araliaceae]	*In Vitro*	THP-1 cells and RA-FLS	0, 12.5, 25, 50, 100 μM	Untreated control	24 h	Regulation of phosphodiesterase 4 (PDE4),blocking the interaction between macrophages and RA-FLS	(Cao et al., 2022b)
				THP-1 cells stimulated with PMA (100 nM) + LPS (100 ng/mL) + IFN-γ (20 ng/mL)	12.5, 25, 50 μM	Control (untreated), M1-polarized only	24 h		
				THP-1 cells (M1-polarized)	12.5, 25, 50 μM	Control, siPDE4B, Apremilast (PDE4 inhibitor)	24 h		
				Co-culture system: M1-polarized THP-1 supernatants on RA-FLS	12.5, 25, 50 μM (on THP-1 cells)	Control supernatants, M1 supernatants only	24 h co-culture		
				Human RA-FLS from synovial tissues	0, 12.5, 25, 50, 100 μM (direct treatment)	Untreated RA-FLS	24 h		
				THP-1 cells stimulated with IL-4 (20 ng/mL)	12.5, 25, 50 μM	Control, M2-polarized only	24 h		
​	​	​	*In Vivo*	Collagen-Induced Arthritis (CIA) SD rats	5, 10, 20 mg/kg/day	Control (saline), CIA model (saline), MTX (0.5 mg/kg)	28 days treatment (day 28–56)	​	​
				CIA rat ankle joints	5, 10, 20 mg/kg/day	Control, CIA model, MTX positive control	28 days treatment		
				CIA rat serum and synovial tissue	5, 10, 20 mg/kg/day	Control, CIA model, MTX positive control	28 days treatment		
				CIA rat synovial tissue	5, 10, 20 mg/kg/day	Control, CIA model	28 days treatment		
				CIA rat synovial tissue	20 mg/kg/day (H-SRG)	Control, CIA model	28 days treatment		
				CIA rat synovial tissue	5, 10, 20 mg/kg/day	Control, CIA model	28 days treatment		
				CIA rat synovial tissues	20 mg/kg/day (H-SRG)	Control, CIA model	28 days treatment		
4	artemisinin(ART)	*Artemisia annua* L.[Asteraceae]	*In Vitro*	RAW 264.7 macrophages (unstimulated)	Gradient concentrations of ART and DEX	Untreated cells, FITC solution, free HA (competitive inhibition)	Up to 72 h (cytotoxicity), 2–4 h (uptake), 24 h (ROS/HIF-1α)	efficiently perform ROS scavenging and repolarization of M1 to M2 macrophages by inhibition of the HIF-1α/NF-κB cascade	(Deng et al., 2024)
				RAW 264.7 macrophages (LPS-stimulated)	Gradient concentrations of ART and DEX	LPS-only, untreated, free HA, FITC solution	Up to 72 h (cytotoxicity), 2–4 h (uptake), 24 h (ROS/HIF-1α), 48 h (apoptosis)		
				RAW 264.7 macrophages (LPS + hypoxia)	Not specified (same as above)	Hypoxia + LPS, untreated	4 h pre-treatment + 4 h hypoxia		
				Cell-free system (ROS-responsive release)	N/A	PBS without H_2_O_2_	24 h		
			*In Vivo*	AIA rat model (Adjuvant-Induced Arthritis)	2.5 mg/kg DEX (equivalent), ART 2.5 mg/kg	Saline, free ART, free DEX, ART&DEX, HAA, HTA, DEX/HAA	29 days (treatment from day 14–29, every 3 days)		
				SD rats (pharmacokinetics)	10 mg/kg DEX	DEX solution	Up to 50 h		
				SD rats (biodistribution)	10 mg/kg DEX, equivalent ART	ART solution, DEX solution	24 h post-injection		
5	Cepharanthine	*Stephania japonica* (Thunb.) Miers[Menispermaceae]	*In Vitro*	THP-1 human monocyte cell line (cytotoxicity)	Various concentrations up to 10 μg/mL	DMSO vehicle	24 h	IL-6-JAK/STAT3 signaling, glycolysis, and oxidative phosphorylation process were downregulated	(Lin et al., 2023b)
				THP-1 cells (chemotaxis assay)	Not specified (doses < 10 μg/mL)	DMSO, MCP-1 (100 ng/mL) or fMLP (100 nM)	3 h (migration)		
				THP-1 derived macrophages (M1 polarization)	2.5–5 μg/mL	DMSO, LPS (100 ng/mL) + IFN-γ (50 ng/mL), IL-4 + IL-13 (M2 control)	24 h (polarization)		
				Mouse Bone Marrow-Derived Macrophages (BMDM)	5 μg/mL	DMSO, LPS + IFN-γ	24 h (polarization)		
				THP-1 derived macrophages (RNA-seq and metabolism)	5 μg/mL	DMSO, LPS + IFN-γ, 2-DG (8.2 mg/mL)	6 h (RNA-seq) or 24 h (component measurement)		
			*In Vivo*	Collagen-Induced Arthritis (CIA) mice (DBA1/J male, 8–10 weeks)	5 mg/kg	Vehicle (PBS), Positive control: MTX (1.5 mg/kg)	18 days (from day 20 to day 38)		
			​	THP-1-derived macrophages	6 mg/mL	M0, M1, M1+EPH, vector + M1, TLR8+M1, TLR8+EPH + M1, siNC, siTLR8	36–48 h		
			*In Vivo*	Wistar rats (FCA-induced RA model)	20–40 mg/kg	Sham, Model, EPH-L, EPH-H	21 days		

#### 3.2.4 Improving the *in vivo* process of TCM metabolites to enhance the therapeutic effect of rheumatoid arthritis treatment

However, some metabolites also have the disadvantage of poor oral absorption, which affects the therapeutic effect. Scientists have also used various methods to alter internal processes in order to enhance drug development, for example, by combining Triptolide with Metformin to combat RA, a novel microenvironment driven deformable self-assembled nanoplatform was constructed using nano drug delivery technology. This platform is assembled from the vascular targeting peptide (STP), Triptolide (TPL), and Metformin (MET). It exhibits excellent dual response characteristics of lesion microenvironment and spatiotemporal grading targeting ability both *in vivo* and *In Vitro*: firstly, it targets VEGFR2, which is highly expressed in the vascular endothelium of RA lesions, through STP peptide, and dissociates under the stimulation of MMP2, which is highly expressed in the lesions, and acidic conditions. The released STP segments self assemble into nanofibers in acidic environment, forming a physical barrier, quickly blocking the infiltration of inflammatory cells, and promoting the normalization of vascular function; Spherical nanoparticles undergo charge reversal and expose the secondary targeting sequence RGD, carrying TPL and MET deep into the joint cavity to precisely target diseased cells, achieving multiple therapeutic effects including immune regulation, anti-inflammatory, and bone protection (Yan et al., 2024). It was found that roburic acid (RBA), a metabolite from anti-RA botanical drug Gentiana macrophylla Pall., displayed strong anti-inflammatory activity. However, its medical application is limited by its hydrophobicity, lack of targeting capability and unclear functional mechanism. Researchers have developed a pH responsive dual target drug delivery system - RBA nanoparticles (RBA NPs), which simultaneously targets CD44 and folate receptors and explores their pharmacological mechanisms. In a rat RA model, nanocarriers effectively deliver RBA to the site of inflammation, significantly improving therapeutic efficacy compared to free RBA, while significantly reducing levels of inflammatory cytokines and promoting tissue repair. Analysis shows that RBA can reprogram M1 macrophages in joints into M2 phenotype. Due to the important role of the balance between pro-inflammatory and anti-inflammatory macrophages in maintaining immune homeostasis and preventing excessive inflammation in RA, this reprogramming may be the key to the anti RA effect. In addition, we found that RBA NPs downregulate glycolysis levels by blocking the ERK/HIF-1 α/GLUT1 pathway, driving the M1 to M2 phenotype transition. Therefore, this study not only developed a targeted delivery system that significantly enhances the anti RA efficiency of RBA, but also revealed potential molecular targets for reversing macrophage reprogramming through energy metabolism regulation.

In summary these research findings illustrate the capacity of TCM metabolites to precisely regulate macrophage polarization states through specific targeting of key signaling pathways, further enhanced their potential as drugs, thereby providing a robust foundation for the development of novel therapeutics for RA

Notably, several metabolites listed above contain PAINS alert substructures. However, all cited original literatures provided cross-orthogonal cellular detection and *in vivo* animal evidence to confirm target-specific regulation of macrophage polarization, eliminating the possibility that the observed anti-inflammatory activity originates from pan-assay interference artifacts (Figure 4).

**FIGURE 4:**
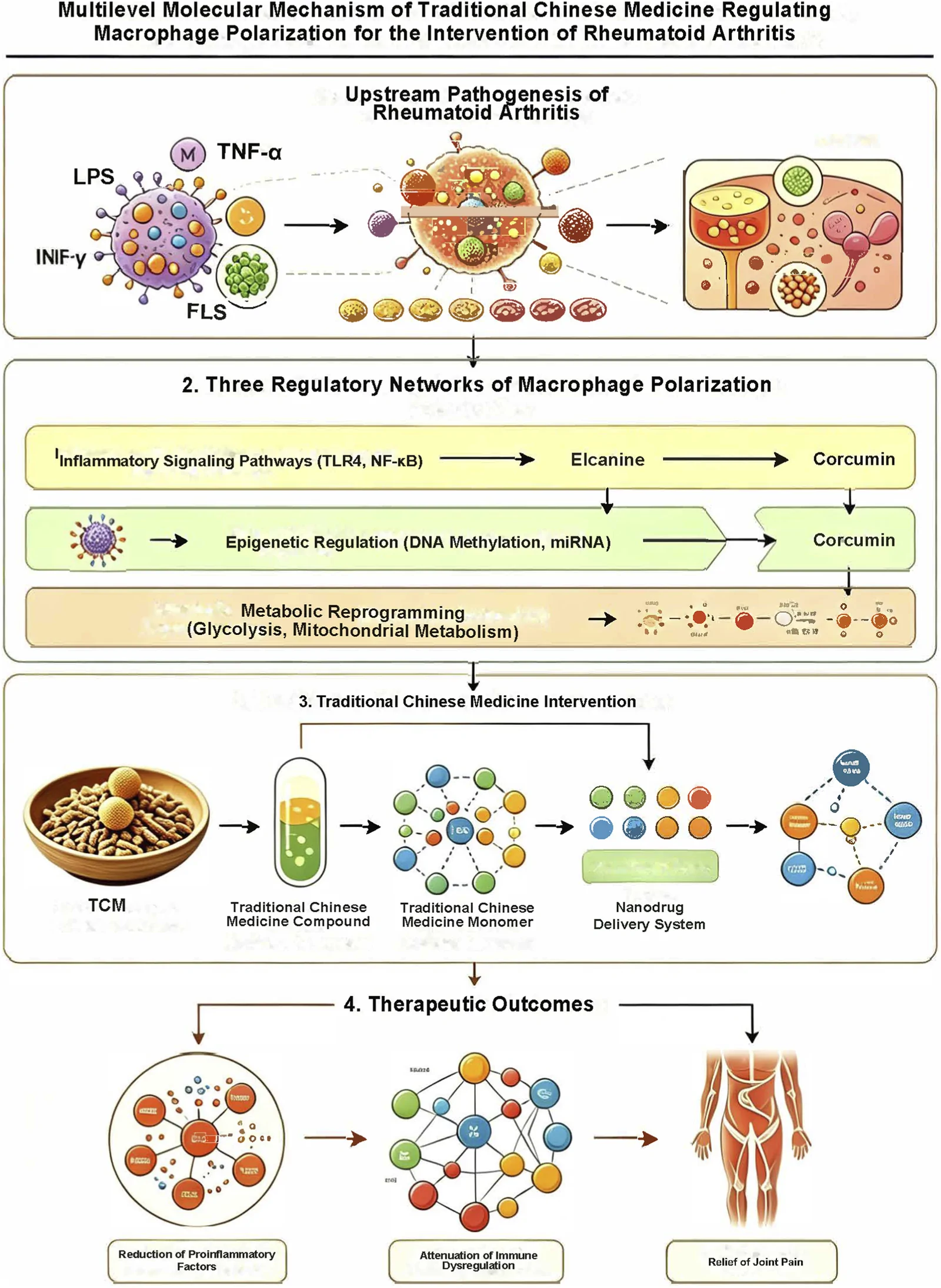
Multilevel molecular mechanism of traditional Chinese medicine regulating macrophage polarization for the intervention of rheumatoid arthritis. The pathological microenvironment induced by inflammatory cytokines drives macrophages toward M1 polarization. The active metabolites derived from traditional Chinese medicine exert synergistic intervention effects. These metabolites inhibit excessive M1 polarization, suppress inflammatory cell infiltration and activation, and thereby achieve anti-rheumatoid arthritis efficacy. Nanodrug delivery technology can improve the targeted enrichment of traditional Chinese medicine metabolites in lesions and enhance the overall therapeutic effect.

## 4 Conclusions and perspectives

TCM—including botanical drugs, compound formulas and active metabolites—demonstrates multi-target and multi-pathway synergistic effects in modulating macrophage polarization for the treatment of RA. Botanical drug formulas such as Liuwei Dihuang Wan primarily regulate key pathways including DNMT, NF-κB, and JAK–STAT, thereby inhibiting the release of pro-inflammatory cytokines (e.g., TNF-α, IL-1β, IL-6) from M1 macrophages while promoting the expression of anti-inflammatory factors (e.g., IL-10, TGF-β) in M2 macrophages. Consequently, these actions ameliorate both inflammation and bone erosion in RA (Gu et al., 2025). Metabolites, including sinomenine and icariin, exert more precise regulatory effects on macrophage polarization through mechanisms such as modulation of the PI3K/AKT pathway, glucose metabolism, and autophagy. Some metabolites also inhibit proptosis or restore Th17/Treg balance (Yan et al., 2024; Zhou et al., 2025).

Modern medical technologies offer robust support for the research and development of TCM. Multi-omics approaches—including transcriptomics, proteomics, and metabolomics—enable in-depth dissection of TCM mechanisms by elucidating molecular-level regulatory networks, thereby providing a scientific basis for precision TCM therapy. For instance, metabolomic analyses of RA patients undergoing TCM treatment can identify alterations in metabolic profiles and reveal modulations of key metabolic pathways, advancing our understanding of TCM’s therapeutic mechanisms (Zou and Zhang, 2022). The functional states of macrophages are distributed along a continuous spectrum, transcending the classic dichotomy of pro-inflammatory (M1) and anti-inflammatory/repair (M2) phenotypes. These states are dynamically shaped by spatiotemporally heterogeneous microenvironmental signals and synergistically regulated through complex molecular networks, with key signaling pathways directing the polarization orientation. The phenomenon of metabolic reprogramming reveals that M1 polarization relies on glycolysis and the pentose phosphate pathway, whereas M2 polarization preferentially utilizes oxidative phosphorylation and fatty acid oxidation. These processes not only supply energy but also generate regulatory metabolites. These mechanisms are interconnected and form feedback loops, collectively shaping the functional diversity of macrophages. Dysregulated polarization is a key pathogenic mechanism of rheumatoid arthritis (RA). Accordingly, we have also identified a shifting trend in research on the regulation of macrophage polarization by traditional Chinese medicine (TCM) for the treatment of RA. Previous studies mainly focused on the modulation of macrophage polarization through inflammation-related pathways (Table 2), as the understanding of macrophage polarization at that time was still limited to the classic M1/M2 paradigm. For example,: inhibiting the activation of the NLRP3 inflammasome in M1 macrophages to reduce the release of inflammatory cytokines (Wu et al., 2023; Gao and Guo, 2024); alleviating inflammation and promoting the polarization of anti-inflammatory M2 macrophages by regulating autophagy (Yang et al., 2024); and restoring the polarization balance of macrophages, among other mechanisms (Zhang et al., 2023).In contrast, current research has extensively and in-depth explored the regulatory network of metabolic reprogramming on macrophage polarization (Table 4), and some studies have further delved into epigenetic regulation (Table 3). These studies will deepen our understanding of the mechanistic network underlying the regulation of macrophage polarization by TCM for the treatment of RA.

The future of TCM in the treatment of RA presents numerous promising avenues for exploration. One critical direction involves the in-depth investigation of classical TCM formulas. Many traditional prescriptions have demonstrated efficacy in long-term clinical practice; however, their mechanisms of action remain incompletely elucidated. Advanced technological approaches—such as systems biology and network pharmacology—offer opportunities to comprehensively analyze the multi-component, multi-target mechanisms underlying these formulas. Such insights could facilitate the optimization of botanical metabolites and enhance therapeutic outcomes. For example, further research into classical anti-RA prescriptions may identify key active metabolites and their targets, enabling the development of more effective and safer TCM-based preparations through rational compatibility optimization and improved preparation processes (Gu et al., 2025).

Another strategic focus is the screening and development of novel anti-RA agents derived from TCM with clearly defined mechanisms and robust efficacy. Bioactive metabolites capable of modulating macrophage polarization represent particularly promising candidates. Through chemical modification and structural optimization, their bioavailability and therapeutic potency could be enhanced, supporting the creation of innovative drugs with independent intellectual property. Concurrently, rigorous safety evaluations are essential to ensure clinical applicability and patient safety. Furthermore, conducting multi-center, large-sample, high-quality clinical trials will be crucial to validate the efficacy and safety of TCM interventions for RA. Such evidence is necessary to support the broader integration of TCM into mainstream RA treatment paradigms (de Molon, 2025).

Furthermore, modern diagnostic and monitoring technologies—such as medical imaging and biomarker detection—can be integrated with TCM syndrome differentia-tion and treatment principles to achieve precise diagnosis and personalized therapeutic strategies for RA patients. By synergizing the strengths of TCM and contemporary medicine, more effective and safer treatment regimens can be developed for RA, ultimately driving progress in the management of this disease.

### 4.1 Supplementary discussion on PAINS risk of TCM metabolites

Many botanical bioactive metabolites summarized in this review carry typical substructural alerts defined by the classic Baell-Holloway PAINS filter, including polyphenols, quinones, α,β-unsaturated carbonyl metabolites. It should be emphasized that the PAINS prediction rules were originally established based on synthetic small-molecule Alpha Screen screening datasets, which produce high false-positive rates for plant-derived natural products.

Unlike synthetic hit metabolites, natural polyphenols, flavonoids and terpenoids with PAINS alerts often exert specific immunomodulatory effects *via* defined signaling axes (TLR/NF-κB, JAK/STAT, HIF-1α glycolytic pathway, epigenetic modification) rather than non-specific pan-assay interference. All original research cited herein adopted multi-layered verification systems to exclude artifactual activity: consistent phenotypic changes were observed across qPCR, ELISA, immunofluorescence, flow cytometry and *in vivo* CIA arthritis animal models; gene knockdown of core target pathways completely abolished the macrophage polarization-regulating effects of these metabolites. These cross-system reproducible observations cannot be explained by aggregation, optical interference or random covalent modification, which are typical PAINS-mediated false positives limited to single *in vitro* biochemical assays.

Moreover, numerous clinically validated anti-inflammatory natural medicines bearing PAINS-warning skeletons have been developed, further indicating that structural PAINS alerts only represent potential risk rather than definitive proof of non-specific artifacts. This review objectively summarizes existing research without overstating the druggability of these metabolites; we also prospectively point out that subsequent structural modification to remove reactive functional groups (excessive catechol hydroxyls, unsaturated electrophilic carbonyls) is a necessary direction to reduce non-specific reactivity and optimize lead compounds from TCM (Bolz et al., 2021) (Supplementary Table S1).
