# Supplementary material for: Advances in traditional Chinese medicine for modulating macrophage polarization in the treatment of rheumatoid arthritis
Source: Front Pharmacol. 2026 Sep 15;17:1702947. doi: 10.3389/fphar.2026.1702947 (PMC13620462; doi:10.3389/fphar.2026.1702947)
Supplement: Supplementary file 1 [file Table1.docx]

S Table 1 PAINS Risk Assessment of Medicinal Plant-Derived Compounds

| **Compound Name** | **Medicinal Plant Source** | **PAINS Alert Structural Type** | **Potential Interference Mechanism** | **PAINS Risk Level** |
| --- | --- | --- | --- | --- |
| Curcumin | Turmeric (Curcuma longa) | α,β-unsaturated diketone (Michael acceptor), catechol polyphenol | Covalent modification of thiol groups, ROS production via oxidation, colloidal aggregation, fluorescence interference | High |
| Punicalagin | Pomegranate (Punica granatum) | Polypyrogallol / catechol tannin | Metal chelation, non-specific protein binding, oxidative stress | Medium-High |
| Icariin | Epimedium (Horny Goat Weed) | Flavonoid with catechol hydroxyl groups | Weak aggregation, mild oxidative interference | Medium |
| Wilforlide A | Thunder God Vine (Tripterygium wilfordii) | α,β-unsaturated lactone | Electrophilic covalent binding to protein thiol groups, broad-spectrum inhibition | Medium-High |
| Aristolochic acid II | Birthwort (Aristolochia) | Nitroarene, catechol hydroxyl group | Oxidative stress, protein alkylation | High |
| α-Mangostin | Mangosteen (Garcinia mangostana) | Polyhydroxy xanthone (quinone derivative) | Quinone redox cycling, non-specific binding | High |
| TFDG (Theaflavin-3,3'-digallate) | Tea (Camellia sinensis) | Polypyrogallol polyphenol | Strong metal chelation, colloidal aggregation | Medium-High |
| Cepharanthine | Stephania tetrandra | Polycyclic aromatic phenol | Fluorescence interference, protein adsorption | Medium |
| Sinomenine | Sinomenium acutum | Phenolic hydroxyl group, cyclohexenone | Slight oxidative interference | Low-Medium |
| Astragalin, Lonicerin, Geniposide | Astragalus, Honeysuckle (Lonicera japonica), Gardenia | Mono-/diphenol flavonoid | Weak aggregation tendency | Low-Medium |
| Artemisinin | Sweet Wormwood (Artemisia annua) | Peroxide bridge | Almost no classic PAINS alerts, weak oxidation effect | Low |
| Roburic acid | Largeleaf Gentian (Gentiana macrophylla) | Monophenol ring | Extremely low interference risk | Low |
